# Proteomic Research of the Stress Response of *Saccharomyces cerevisiae W303* Yeast to Metal Ions Eluted from Orthodontic Appliances

**DOI:** 10.3390/microorganisms13092200

**Published:** 2025-09-19

**Authors:** Lara Dežulović, Božena Ćurko-Cofek, Gordana Čanadi Jurešić

**Affiliations:** 1 Luka Rijeka d.d., 51000 Rijeka, Croatia; ldezulov@gmail.com; 2 Department of Physiology and Immunology, Faculty of Medicine, University of Rijeka, 51000 Rijeka, Croatia; bozena.curko.cofek@medri.uniri.hr; 3 Department of Medical Chemistry, Biochemistry and Clinical Chemistry, Faculty of Medicine, University of Rijeka, 51000 Rijeka, Croatia

**Keywords:** proteomic research, stress response, yeast, metal ions, orthodontic appliances

## Abstract

Although orthodontic appliances are widely used in daily practice, they also have their downsides due to the large amount of metal ions released from their surface. In this study, the influence of such released metal ions on the yeast *Saccharomyces cerevisiae* W303 as a model organism was investigated. Experimental yeast media in which metal ions (iron, aluminum, nickel, chromium, copper, and manganese) were eluted for 3, 7, 14, and 28 days were prepared and then used for yeast cultivation (up to the early stationary growth phase). The growth, increase, and viability of the cells were tested. The mitochondria were isolated from the spheroplasts, and the mitochondrial proteins were obtained and analyzed by liquid chromatography/mass spectrometry. Fortythree significantly altered proteins were identified. They showed significantly reduced expression in all metal-treated groups compared to the control. The metabolic processes for energy supply (glycolysis, gluconeogenesis, tricarboxylic acid cycle, and adenosine triphosphate synthesis) dominated with 50% of the total amount of significantly altered proteins in all samples, but their proportions changed at different time points. The downregulation of mitochondrial proteins such as Atp1, Atp2, and Pet9 under conditions of metal overload suggests a broader impairment of mitochondrial function. Three levels of response to stress can be observed—at relatively low metal ion concentrations in the medium (3 days of elution, approx. 3 mg/L), at medium concentrations (7 days of elution, approx. 5.5 mg/L), and at high concentrations (>8 mg/L, 14 and 28 days of elution), each affecting a specific group of proteins. The results show that mixtures of metal ions in experimental media led to a specific response (in terms of the amount and type of proteins) in each sample type to combat the provoked stress.

## 1. Introduction

Metals are abundant in nature and are essential components of all living organisms. The ions of transition metals play a key role in various biological processes. Therefore, the essential metals (such as iron (Fe), copper (Cu), manganese (Mn), nickel (Ni), and zinc (Zn)) must be present in trace amounts in living organisms to ensure the normal course of biochemical processes. However, in excessive amounts, they can be harmful due to overexposure or genetic mutations that inhibit intracellular regulatory processes and disrupt cellular homeostasis [1]. Non-essential metals, such as cadmium (Cd), chromium (Cr), lead (Pb), arsenic (As), and mercury (Hg), can bind to cellular biomolecules and interfere with physiological cellular processes. Thus, it can be said that high concentrations of metal ions are harmful to living organisms [2]. Namely, unlike toxic organic compounds, metals cannot be degraded or modified and remain in cells, interfering with cellular homeostatic pathways. Metal toxicity at the cellular and organ levels can result from oxidative stress (OS), inhibition of enzyme function, disruption of protein function, lipid peroxidation, DNA damage, and apoptosis [3]. To maintain the balance between essential and toxic levels of metals, cells use protective mechanisms to regulate uptake and detoxification, thus preserving cellular and genetic integrity. Any disturbance of the detoxification system or metal homeostasis can cause several human diseases, including cancer.

Many metals can cause the phenomenon of oxidative stress (OS) [4]. OS is a metabolic state of the organism associated with an increase in the amount of reactive oxygen species (ROS) and a disturbance of the balance between oxidants and antioxidants in favor of the oxidants. Free radicals of oxygen (superoxide anion radical (O^2−.^), hydrogen peroxide (H_2_O_2_), hydroxyl radical (∙OH), singlet oxygen (^1^O_2_)) are produced during cell metabolism by aerobic organisms and exert some beneficial effects on the body under normal conditions [5]. Every organism has an adequate antioxidant defense system to cope with excessive ROS production and maintain redox homeostasis. When an excess of ROS is present, the cell’s antioxidant defense is insufficient and results in damage to cellular components, such as membranes, lipids, proteins, and nucleic acids. Severe damage to these biomolecules can lead to cell death [6,7,8].

In order to effectively study the harmful effects of OS on the human body, it is necessary to use a model organism that accurately represents the events in the organism it replaces [9]. One of these model organisms is the yeast *Saccharomyces cerevisiae*, a unicellular microorganism with excellent properties for research at the cellular and molecular level. It is suitable for studying the effects of metal toxicity on biological processes and to identify similar mechanisms in other organisms [4]. The proteins of *S. cerevisiae* are homologous to human proteins, making yeast an effective research model. About 30% of the genes involved in human diseases may have orthologs in the yeast proteome [9]. Despite its simplicity, *S. cerevisiae* is at the forefront of genomics research as it enables the understanding of the molecular mechanisms and functional pathways involved in the cellular response to toxic substances. Many mechanisms involved in the transport and homeostasis of metal ions have been elucidated using *S. cerevisiae* [10]. In addition, the yeast *S. cerevisiae* was used in research on oxidative stress-induced programmed cell death. It has been discovered that in response to accumulated ROS, transcriptional changes (with upregulated antioxidants), activation of mitophagy as a pro-survival pathway, and programmed cell death (with autophagy, necrosis, and apoptosis) occur [7,8].

Nowadays, metal devices are frequently used in various medical fields, including cardiovascular medicine, orthopedics, otorhinolaryngology, and dentistry. They are implanted into the patient’s body to treat and correct defects; therefore, their biocompatibility is of utmost importance. Their ideal properties are jeopardized by the occurrence of corrosion and potentially unfavorable local tissue reactions [11]. Released metal ions can bind to biomolecules and cause OS. The fixed orthodontic appliances, which consist of various metal parts such as brackets, bands, archwires, mini-screws, and clips, can be used as an example to explain this process [6]. Over time, the oral environment, with its constant changes in pH, temperature, and biological and enzymatic composition, affects the stability of the components of the orthodontic appliance, which tend to corrode and biodegrade. Electrochemical corrosion reactions on the surface of biomaterials are redox reactions in which surface metals are oxidized to metal cations and metal oxides, which in turn can be released into the oral cavity. Saliva has a particular effect on metallic materials because it is an electrolyte [12,13]. The released metal ions fluctuate in the extracellular environment. Each cell must perceive this concentration and act accordingly by establishing a balance between the amounts required for vital cell processes and potentially toxic amounts. In thorough research made by Aulakh et al., all types of *-omic* responses to perturbations in metal availability along concentration gradients of the most important growth-essential metal ions (calcium (Ca), Cu, Fe, potassium (K), magnesium (Mg), Mn, sodium (Na), Zn) to *S. cerevisiae* yeast cells were tested. Although the response of the biological network to each metal is different, a metal homeostasis for each metal (which varies between metals) and an interdependence of the various metal ion concentrations were observed [14].

Previous studies on the toxicity of metals using *S. cerevisiae* as a model indicate the possible negative effects of metal ions. The acute toxicity of Cu, Pb, and Ni metal ions on yeast cells has been demonstrated. The ratio of their toxicity is in the order Cu^2+^ > Pb^2+^ > Ni^2+^ [15]. Yeast cells exposed to Pb showed mutations at the mitochondrial deoxyribonucleic acid (DNA) level [16]. It has been shown that Cu and Mn can induce apoptosis at moderate concentrations by affecting the mitochondria of yeast cells. A sharp increase in the concentration of the metal ions Cu^2+^ and Mn^2+^ can also cause necrosis [17]. Since most toxic metal ions can induce OS in cells via the mitochondrial respiratory chain, mitochondria may both contribute to and serve as targets of this toxicity. As semi-autonomous organelles, mitochondria are a hallmark of eukaryotic cells and are essential for their viability. Mitochondria are composed of two membranes that maintain structural integrity and enable the generation of a proton motive force (PMF) that drives adenosine triphosphate (ATP) synthesis. Mitochondria are considered the center of many significant metabolic processes and, as such, are closely linked to other cellular functions and pathways that occur in separate cellular compartments. In particular, they are dynamic and interconnected organelles that continuously undergo fusion and fission processes to form a tubular transitional structure known as the mitochondrial network. As mitochondria are the site of cellular respiration, i.e., the powerhouses of the cell, they are strongly affected by changes in cellular growth conditions [18,19]. Hence, the rapid growth of yeast cells induces a shift in the cell’s metabolism. Glucose is used as a source of carbon and triggers a significant remodeling of mitochondrial metabolism as well as the shape, structure, and volume of the mitochondrial network. More importantly, this metabolic transition happens before the changes in the rest of the cell, suggesting a higher possibility of determining the early metabolic changes in the cell at the mitochondrial level [19]. High concentrations of silver (Ag^+^) have been shown to induce morphological changes in mitochondria after relatively long exposure [20]. Hosiner et al. investigated the acute toxicity of 10 biologically relevant metals. Cd proved to be the most toxic, followed by Hg, Ag, As, cobalt (Co), aluminum (Al), vanadium (V), Ni, Mn, and Zn. It was also found that about 15% of yeast transcripts changed the expression within 30 min, and that protein degradation is involved in the detoxification process. Re-synthesis of proteins is activated after continuous metal exposure, indicating transcriptional adaptation of yeast cells during prolonged exposure [3]. Therefore, the response of yeast cells to metal exposure in overloaded conditions has been reported as arrest of cell cycle progression and adaptation of the transcriptome, proteome and metabolome [21]. Cells face stress at different time scales and trigger the basic stress response as a reaction to shock [22]. To adapt and to optimize growth and survival during stress, significant changes to the cell’s proteome are required. Reprogramming of gene expression during stress usually involves an initial global repression of protein synthesis, followed by activation of stress-responsive messenger RNAs (mRNAs) through translational and transcriptional responses, and protein degradation [23].

Recent studies emphasize the importance of careful selection of suitable culture media in metal toxicity experiments and the measurement of metal concentrations in culture media [12,14]. Namely, the presence of extensive buffering capacities of cells for metal ions is the reason that changes in metal ion concentrations in the media do not directly translate into proportional changes in cellular metal concentrations. Furthermore, metal ion transporters in living organisms are able to interact with more than one metal ion, and a change in the concentration of one metal ion may have an unintended effect on the cellular concentrations of other metal ions [14]. Regarding the choice of culture media, artificial saliva or minimal media (e.g., without amino acid supplements) or yeast broth with at least 2% glucose as a primary energy source or yeast broth with suppressed glucose content will induce a completely different response of the yeast cells to the metal ions present in the media [12,14,24,25].

One of the most common potential sources of metal ions is fixed orthodontic appliances made from alloys of stainless steel (SS), nickel–titanium (Ni-Ti), cobalt–chromium (Co-Cr-Ni), and β–titanium (Ti-Mo), which consist of a mixture of metals present in a specified percentage. The most common metals are Ni, Cr, Co, Fe, Ti, and Mo. Ni is described as the most allergenic metal in orthodontic alloys, followed by Cr. Orthodontic appliances are generally safe to use as the concentrations of released metal ions do not exceed acceptable limits. Cytotoxicity and oxidative stress induced by metal ions are possible at extremely high concentrations of metal ions (1000 µM). However, in yeast strains without oxidative defenses, the released metal ions from the SS and Co-Cr alloy can induce cytotoxicity and oxidative stress even at lower concentrations (100 µM). This indicates that in patients with a deficient antioxidant defense system, increased ROS formation can occur in the oral cavity even at moderate concentrations [11]. Due to changes in the activity of antioxidant enzymes, there is also the possibility that even non-toxic concentrations of metal ions have biological effects on oral cells, considering hypersensitivity to metals, especially Ni [26]. Ions of Fe, Cr, Mn, and Al (eluted from NiTi archwires and SS brackets, ligatures, and bands) showed a slight pro-oxidant effect on DNA and slight genotoxicity on gastrointestinal cell lines. Under normal conditions, the human body can “resist” these effects [27]. Although there is evidence that constant salivary flow is likely to dilute metal ions released from the orthodontic appliances, it has been shown that metal ions accumulate in deposits on the surface of brackets and wires and, if not removed, can cause local adverse effects [28].

In an attempt to determine the response of yeast to various amounts and types of metal ions present in the culture medium (and eluted by orthodontic appliances), the proteome of mitochondria and related proteins metabolically bound to mitochondria of the yeast *Saccharomyces cerevisiae* W303 was analyzed. Viability assays were used to determine the proliferation of yeast cells in media containing eluted metal ions and the proportion of apoptotic and necrotic cells growing on these media. Four different growth media were used for the study. They were obtained after 3 (3D), 7 (7D), 14 (14D), and 28 days (28D) of elution of the complete orthodontic appliance in complete yeast growth medium, showing progressively increasing concentrations of metal ions released from the appliance (Al, Cr, Cu, Fe, Mn, and Ni) with longer elution times. The experimental medium consisted of yeast growth media containing 2% glucose at pH 5.5, which inherently also contained stable concentrations of metal ions originating from the medium itself (e.g., calcium, magnesium, sodium, potassium). The control group consisted of the same growth medium without appliance-derived metal ions. The presence and content of mitochondria-associated proteins, which are part of the OS reaction, were analyzed in the crude mitochondrial fraction. Biologically important mitochondrial proteins that were differentially expressed in the analyzed samples were identified, clustered by function, quantified, and compared between samples. In addition, we sought to identify specific proteins that may contribute to elucidating the mechanism of the stress response when metal ions, both essential and (cyto)toxic, are present in the growth media, in addition to all essential nutrients, both in minimal and excessive amounts.

## 2. Materials and Methods

### 2.1. Experimental Organism

*Saccharomyces cerevisiae* W303 (MATa/MATα {*leu2-3,112 trp1-1 can1-100 ura3-1 ade2-1 his3-11,15*} an auxotrophic mutant for Ura that is highly sensitive to pH in the presence of metal ions in media, is used in the study [29]. This yeast was kindly provided by the Collection of Microorganisms of the Institute of Molecular Biotechnology, University of Technology, Graz.

### 2.2. Experimental Growth Media

To prepare eluates of orthodontic appliances in yeast peptone dextrose (YPD) media, all parts of a complete orthodontic appliance (two archwires, twenty brackets, twenty ligatures, and four bands) were immersed in 28.0 mL of YPD media (complete yeast growth medium) and autoclaved together. After autoclaving at 121 °C for 15 min (CertoClav, Leonding, Austria), incubation was performed under sterile conditions on a rotary shaker (37 °C, 100 rpm, Unimax 1010, Heidolph, Schwabach, Germany) for 3, 7, 14, and 28 days. The volume of the medium was calculated on the basis of 1 mL per cm of archwire (2 wires of 14 cm). YPD is a mixture of yeast extract (10 g/L, Sigma, St. Louis, MO, USA), peptone (20 g/L, Sigma, USA), and dextrose (glucose, 20 g/L, Fluka, Neu-Ulm, Germany). The pH was adjusted to 5.5 with HCl.

The metal ion concentrations in experimental YPD media (3D, 7D, 14D, 28D eluates and YPD media as control) were measured with an inductively coupled plasma mass spectrometer. In all experimental media, nine ions were selected for monitoring—Al, Co, Cr, Cu, Fe, Mn, Mo, Ni, and Ti—as they were either known constituents of orthodontic appliances, showed changes in concentration during elution, or were not declared components yet were nonetheless detected. The results were presented in our previous publication (as model 2, YPD) [12]. The amount of eluted metal ions increased with the duration of the elution time. The highest concentration of eluted ions (with more than 11 mg/L) was obtained in the 28D elution sample. Iron predominated (with >8 mg/L), followed by Al and Ni, and then Cr and Cu ions. Table 1 shows an abbreviated version of the results presented in the paper, which are necessary for understanding the changes in the proteome in a particular type of sample.

### 2.3. Determining the Vitality of Yeast

The Luna cell counter (LUNA-FL^TM^ Dual Fluorescence cell counter, Logos Biosystems, Anyang, Republic of Korea) was used to determine the cell number at the beginning and end of the experiment in the control and all experimental samples. The start of the experiment was set at approximately 5 × 10^6^ cells/mL. The yeast was pre-cultured in YPD medium for 24 h at 28 °C and then batch cultured in experimental YPD media for 20 h at 28 °C and 160 rpm (until the cells reached early stationary phase, which was determined experimentally by growth curves). The result (as an increase in cell number) is expressed as the ratio between the cell number at the end and the beginning of each experiment in each sample. At least six independent experiments were performed for each sample type.

After each experiment, the vitality of all yeast cells was also determined. For this purpose, an acridine orange/propidium iodide (AO/PI, by Carl Roth, Karlsruhe, Germany) yeast staining was performed. In brief, the freshly prepared dye mixture was added to the yeast suspension (final concentration of dye: 0.3 μg/μL PI and 0.5 μg/μL AO). After 10 min of incubation, centrifugation (3000 rpm for 5 min, Rotina 380, Hettich, Beverly, MA, USA) was performed, and the supernatant was removed. Then, 4% (g/mL) paraformaldehyde was added to each sample and incubated for 15 min at room temperature. After another centrifugation at 3000 rpm for 5 min, the supernatant is removed. The precipitate is washed once in phosphate buffer with the addition of sorbitol (2M sorbitol in 1M KH_2_PO_4_/K_2_HPO_4,_ pH 5.5) and resuspended in a small volume of the same buffer. Within a tested culture, we counted the number of red, orange, and green fluorescent cells and determined the percentage of late apoptotic and necrotic cells in the total number of cells. Apoptotic death shows nuclear condensation and plasma membrane blebbing, while necrotic death is accompanied by visible membrane ruptures [30]. These cells were analyzed from at least 250 cells in every biological replicate. PI only penetrates cells with damaged membranes. Therefore, dying, dead, and necrotic nucleated cells stained with PI produce red fluorescence. AO is a cell-permeable dye, and all living, metabolically active stained nucleated cells produce green fluorescence. An Olympus BX51 fluorescence microscope (Olympus, Tokyo, Japan) was used for microscopic analysis, and ImageJ software (version 1.53t, National Institutes of Health, Bethesda, MD, USA) for image processing.

### 2.4. Preparation of Mitochondrial Protein Samples for Liquid Chromatography/Mass Spectrometry

The isolation of mitochondria begins with the cultivation of yeast cells in experimental YPD media (3D, 7D, 14D, 28D eluates and YPD media as control) overnight at 30 °C on a rotary shaker at 200 rpm (Unimax 1000, Heidolph, Schwabach, Germany). Cultivation was performed in three biological replicates. When the cells had reached the early stationary phase, the contents of the flasks were centrifuged (3000 rpm for 5 min), and the prepared cell pellets were used for mitochondria isolation. Mitochondria were isolated using the Yeast Mitochondria Isolation Kit (Abcam ab178779, Cambridge, UK), following the manufacturer’s instructions Briefly, yeast were harvested, washed, and subjected to spheroplast formation using Buffer A supplemented with 10 mM dithiothreitol (DTT) (10 min, 30 °C), followed by Buffer B with Lysis Enzyme Mix (10–15 min, 30 °C), with efficiency of spheroplast formation confirmed by a 30–40% drop in OD_600_. Spheroplasts were homogenized on ice in Yeast Homogenization Buffer containing protease inhibitors (10–15 strokes in a Dounce homogenizer). The homogenate was centrifuged stepwise at 600 rpm to remove debris. In the final step of mitochondrial preparation, the collected supernatant is centrifuged at 12,000 rpm for 10 min at 4 °C. The resulting precipitate is resuspended in 50 μL of storage buffer. The protein concentration (Pierce™ Rapid Gold BCA Protein Assay Kit, Thermoscientific, Waltham, MA, USA) was determined in each sample solution. In this step, all isolated mitochondrial proteins were combined for each sample type, and after centrifugation, precipitation buffer (10% trichloroacetic acid in acetone +20 mM DTT) was added to the resulting precipitate of isolated mitochondria. Protein extraction took place overnight in the cold. The samples were then centrifuged at 3000 rpm, and the resulting precipitate was dissolved in a solution of 5 M urea in 50 mM ammonium bicarbonate. The amount of protein in these samples was also determined using the same kit (Pierce™ Rapid Gold BCA Protein Assay Kit, Thermoscientific). For all samples, the protein concentrations were set to the same value at which the further procedure was initiated. The dilution is performed with a solution of 5 M urea in 50 mM ammonium bicarbonate.

### 2.5. Peptide Analysis with the ESIqTOF Mass Spectrometer

Mitochondrial protein samples were analyzed using the ESI-Q-TOF Synapt G2-Si high-resolution mass spectrometer (Waters, Milford, MA, USA) coupled to the nanoACQUITY UPLC liquid chromatograph (Waters, Milford, MA, USA). The instrument parameters are set using MassLynx software version 4.1. SCN902 (Waters, Milford, MA, USA). Peptides were separated on a nanoAcquity UPLC 1.7 μm BEH130 C18, 100 μm × 100 mm analytical column (Waters, Milford, MA, USA) with a 2G-V/M Trap Symmetry C18 column (5 μm, 180 μm × 20 mm, Waters, Milford, MA, USA). Additionally, 1 μL of the sample was injected, and all samples were analyzed in triplicate. The conditions of a 65 min gradient flush applied for optimal separation of tryptic peptides by reversed-phase chromatography are described in Table 2.

The flow rate was 1 μL/min, and the column temperature was 40 °C.

Electrospray ionization (ESI) was positive, with a capillary voltage of 4.3 kV and a cone voltage of 40 V. The source temperature was 80 °C. The nitrogen pressure was 1.4 bar. The spectra of the peptide samples were collected at low and high energy using the MSE approach. Under low energy conditions, data were collected at a constant collision energy of 4 eV, while under high energy conditions, the collision energy was increased linearly from 20 to 45 eV. Data was collected every second in the range of masses 50–3200 Da. A continuous lock mass correction was performed using leucine encephalin ([M + H]^+^ = 556.2771 Da, 1 ng/μL at 0.4 μL/min).

### 2.6. Identification of Proteins

Raw data was processed and analyzed using ProteinLynx Global Server Software (PLGS; version 3.0.1; Waters). The settings for peak processing were as follows:

- threshold for low energy—135 counts;
- threshold for increased energy—30 counts;
- intensity threshold—750 counts.

The data were searched in the database for the reference proteome *Saccharomyces cerevisiae* (ATCC strain 204508/S288c) (Baker’s yeast) UP000002311. A sequence of trypsin (*Sus scrofa*) was also added to the base with which the protein was digested. The confidence limit was ≥95%. Search settings included up to two missed trypsin cleavages; oxidation of methionine was included as a variable modification. Mass tolerance was set to 10 ppm for precursor ions and 0.02 Da for fragment ions. Protein identification requires at least three ion fragment matches per peptide and seven ion fragment matches with at least two peptide matches. Strict scoring thresholds were applied, with a minimum score of 20 for peptide identification and 50 for protein identification. The false discovery rate (FDR) was controlled using the target-decoy approach, with a threshold set at 1%. Additionally, Percolator was used for re-evaluation of peptide-spectrum matches to ensure stringent scoring criteria. The FDR was estimated at both the peptide and protein levels, ensuring that the reported identifications meet the 1% FDR threshold. 

Expression analysis was performed using a digest with trypsin and bovine serum albumin (BSA) (P02769) as an internal standard at a concentration of 72 fmol/μL.

### 2.7. Analysis of the Proteomic Results

The bioinformatic analysis and visualization of the proteome data (LFQ intensities obtained with PLGS) was performed using Perseus software (version 2.0.11). In the analysis, the differences between four different samples (3D, 7D, 14D, 28D) and YPD media as a control, were plotted. Each sample and control comprised three replicates. To determine the variations and statistical differences between the proteomes of two or more samples, statistical tests were used. Basically, an ANOVA with Tukey post hoc test was used to determine significantly altered proteins, while volcano plots and hierarchical clustering were used for different representations of the obtained results. The volcano plot is a type of scatter plot (two-dimensional *t*-test) that shows the statistical significance (*p*-value) in relation to the extent of the change. It is commonly used in proteomics to identify changes between samples and provide an effective means of visualizing the direction, magnitude, and significance of changes in protein expression.

Hierarchical clustering was used to determine the patterns of protein expression and visualize them in a simple way as a heat map.

Additionally, absolutely quantified PLGS data were used to support and validate the Perseus analysis and to generate graphs of the relative proportions of detected proteins (Figure 3d).

### 2.8. Statistical Analysis

The results of the vitality tests were analyzed using the Statistica software package (TIBCO Statistica, version 13.4.0.14) (Man–Whitney U test, *p* < 0.05).

## 3. Results

This study investigated how yeast responds to stress caused by different amounts and types of metal ions eluted from orthodontic appliances. In contrast to most studies carried out [3,13,26,31,32], we achieved exceptionally high concentrations of eluted metal ions in the media tested in this study. In addition, the medium used was a yeast broth, a protein-containing sugary medium that stimulated an extremely high release of metal ions, as no compact protective layer could form on the surface [12].

**Figure 1:**
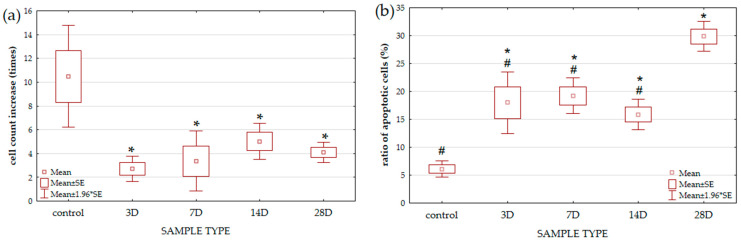
Determination of yeast cell viability and vitality. Viability of yeast cells (**a**) is expressed as the increase in cell number (the ratio between the cell number at the end and at the beginning of the experiment) of each sample. Vitality of yeast cells (**b**) is expressed as the percentage of late apoptotic and necrotic cells in relation to the total number of cells. Samples after 3D, 7D, 14D, and 28D elution and YPD media as control were used for the study. The total number of samples was 9 for control, 8 for 3D, 6 for 7D, 8 for 14D, and 8 for 28D. Results are expressed as mean + SE (Man-Whitney U test, the level of statistical significance is *p* < 0.05). * significantly different from control (* *p* < 0.05); ^#^ significantly different from 28D sample (^#^ *p* < 0.05).

At the beginning of any toxicity test, in addition to vitality and metabolic activity, it is useful to perform simple tests such as growth curves and changes in the number, size, and growth mode of cells under experimental (altered, inappropriate, or even toxic) conditions. In this work, a growth inhibition test was performed. Figure 1a shows that all cells growing on media with eluted metal ions (3D, 7D, 14D, and 28D) exhibit significantly reduced cell growth compared to the control sample.

Athough there are differences between the treated samples, and a trend of increased growth from the 3rd to the 14th day can be observed, there are no significant differences between the treated samples. In this work, the metabolic activity was examined by AO/PI staining. Dying, dead (with nuclear condensation) and necrotic yeast cells (with visible membrane ruptures), Ref. [30] stained red with PI, were counted in at least 1000 analyzed cells per sample. Figure 1b shows that treated samples contain many necrotic and apoptotic cells. In samples 3D to 14D, there is a negative correlation between an increase in cell number and a decrease in apoptotic and necrotic cells. Sample 28D deviates from this correlation and differs significantly from all other samples in the (higher) amount of necrotic and apoptotic cells.

**Figure 2:**
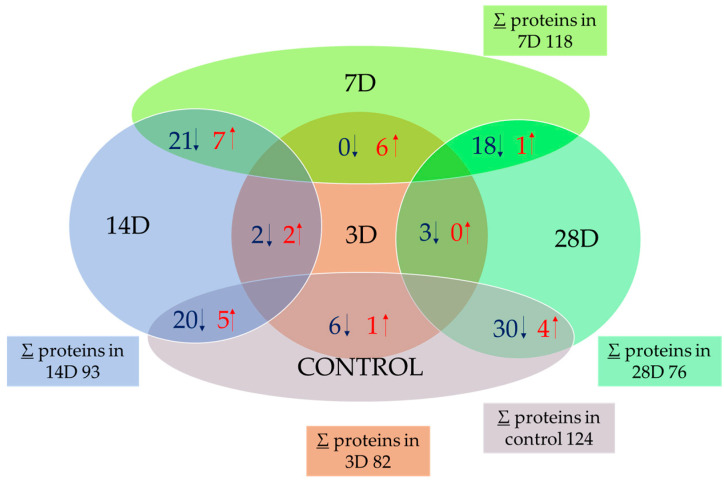
Total number of isolated mitochondrial proteins and number of overlapping proteins, represented by the Venn diagram. The total number of proteins identified was the mean of all proteins identified by PLGS within each sample type (∑). Overlapping numbers are determined by the volcano plot test (given in Supplementary S1). Samples after 3D, 7D, 14D, and 28D elution and YPD media as control were used. The values in overlapping regions are given either as a relation to the control or as a higher number of elution days compared to a lower number (e.g., 28D vs. 3D). Red arrows (↑) indicate the number of proteins significantly increased in the respective comparison, while blue arrows (↓) indicate the number of proteins significantly decreased.

To investigate the changes that occur in the cell, we focused on the identification of mitochondrial proteins that changed expression, as well as on the changes in their amounts at different times of exposure. We obtained quantitative data for 236 proteins using a mass spectrometer. From this original number, we used a multidimensional ANOVA test to identify 43 proteins that showed significant differential expression in the experimental samples used (3D, 7D, 14D, and 28D). The changes were statistically significant at 95% (*p* < 0.05). The total number of mitochondrial proteins isolated and identified by sample type by PLGS is shown in Figure 2. Significantly different proteins (calculated by the two-sample T-test and visualized with a volcano plot) are presented in overlapping parts of the samples. The values of total proteins decrease in the treated samples compared to the control (∑ = 124). The smallest change in the number of proteins was observed in sample 7D (∑ = 118), while the lowest number of proteins was identified in sample 28D (∑ = 76). The comparison between the samples shows no mutual difference between the control and sample 7D, and no mutual difference between samples 14D and 28D. On the other hand, 14D and 28D are most different from the control and 7D. Three-dimensional is similar to 14D and 28D and differs only slightly from them (only a few proteins were decreased or increased).

**Figure 3:**
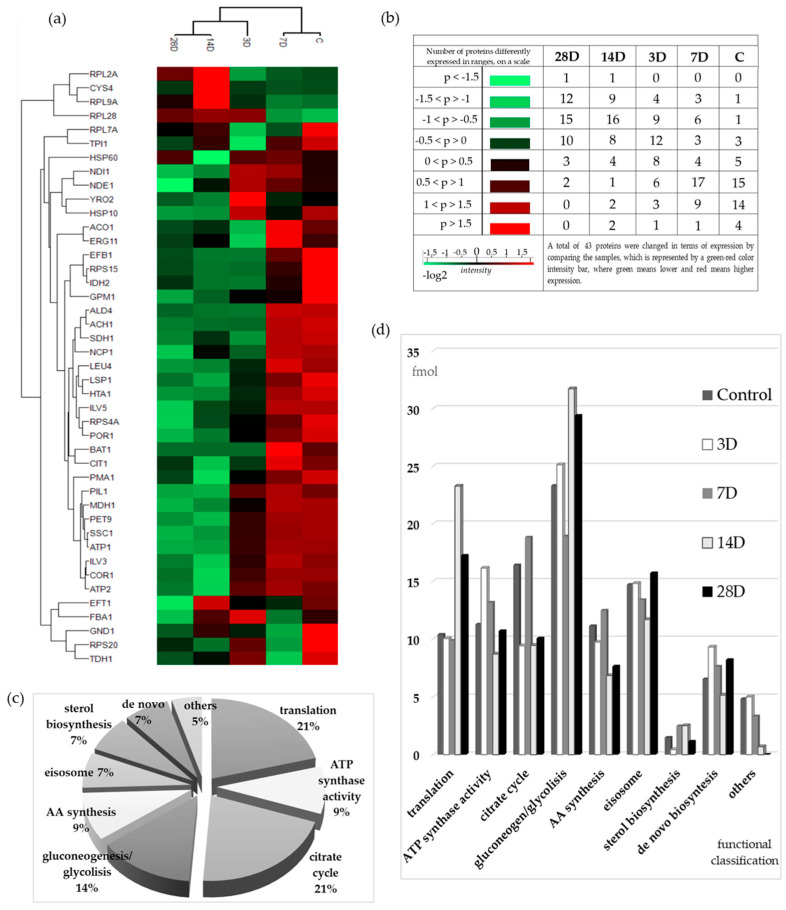
The hierarchical clustering of mitochondrial proteins. (**a**) Analysis of the protein expression pattern presented by a heat map. Red color represents increased expression, while green color shows decreased expression. The higher the value, the more intense the color. (**b**) Number of differentially expressed proteins by range of Log2 values, distributed by sample type. (**c**) Functional classification (made by String database) of 43 significantly changed proteins upon treatment with experimental media (orthodontic appliances eluates samples after 3 days (3D), 7 days (7D), 14 days (14D), and 28 days (28D) of elution and YPD media as control) expressed as a relative proportion of all identified proteins. (**d**) Relative molar proportions of the proteins classified by function, calculated from the data of the PLGS expression analysis, which was carried out using a digest with trypsin and BSA as internal standard (72 fmol/µL).

The hierarchical clustering (Figure 3a,b) was based on the ANOVA (multiple-sample *t* test) calculation, and the proteins that reached the significance threshold were highlighted. A permutation-based FDR was used to control false-positive results in a series of rejected hypotheses at a significance level of 0.05. Using the ANOVA test, we identified 43 significantly different proteins, and with Tukey’s post hoc test, the difference between the groups was determined.

The values were displayed two-dimensionally using a dual color scale—red indicating increased and green decreased expression—where the intensity of color reflects the magnitude of change. The more extreme the value, the more intense the color (lighter shade). This heat map shows two dendrograms representing the result of the clustering on two levels, i.e., on the level of the samples (columns) and on the level of the proteins (rows). The samples and proteins were grouped based on the similarity of their expression patterns. The control was grouped with the 7D, 14D, with 28D samples, while the 3D sample was in the middle, more related to the 14D and 28D samples. A more detailed examination of the heat map and the clustering of proteins often requires exploring deeper biochemical mechanisms to understand the reasons behind such groupings. For example, in the top cluster (Figure 3a), containing large ribosomal subunit proteins (Rpl2A, Rpl9A, and Rpl28), the protein Cys4 is also included. Cys4 plays a key role in cysteine biosynthesis, which is directly linked to glutathione production—a major cellular antioxidant involved in the response to OS and detoxification of metal ions [33]. In that cluster, nearly all proteins show their highest expression levels in sample 14D, making this cluster particularly distinctive. It is also worth highlighting the cluster formed by Efb1, Rps15, Idh2, and Gpm1. Although these proteins have different cellular functions—Efb1 (translation elongation), Rps15 (ribosomal structure and protein synthesis), Idh2 (mitochondrial energy metabolism), and Gpm1 (glycolysis)—they were clustered together due to the same expression trend, showing the highest expression in the control (*p* > 1.5) and reduced expression in all treated samples [34]. Another interesting cluster includes ATPases (Atp1 and Atp2), Pil1, Mdh1, Pet9, and Ssc1. Pil1, a component of eisosomes, is involved in plasma membrane organization and endocytosis [35], whereas Mdh1 participates in the tricarboxylic acid cycle, Pet9 functions as the ADP/ATP translocase, and Ssc1 acts as a mitochondrial Hsp70 chaperone assisting protein import into mitochondria [34]. Atp1 and Atp2 are subunits of the mitochondrial ATP synthase complex, essential for oxidative phosphorylation and energy production [18]. The hierarchical clustering pattern and color distribution reveal a general trend: the control sample and 7D exhibit predominantly higher expression levels (red), while the remaining treated samples show reduced expression (green), more pronounced in either 14D or 28D (lighter green). The 3D sample shows expression levels around zero (dark color). This clustering suggests that, despite different molecular functions and localization in distinct cellular compartments, these proteins follow a similar trend of decreased expression under metal exposure, further indicating a coordinated interplay among organelles in the fine regulation of metabolism.

Figure 3b presents the number of differentially expressed proteins by Log2 value and sample type. For proteins with Log2 values < 0, the totals were 38 (28D), 36 (14D), 25 (3D), 12 (7D), and 5 (control). For proteins with Log2 values > 0, the corresponding totals were 5, 9, 18, 31, and 38 for the same sample order. Notably, the highest number of downregulated proteins was observed in the sample with the highest amount of eluted metal ions in the medium, suggesting that this level of metal exposure strongly affects protein downregulation.

**Figure 4:**
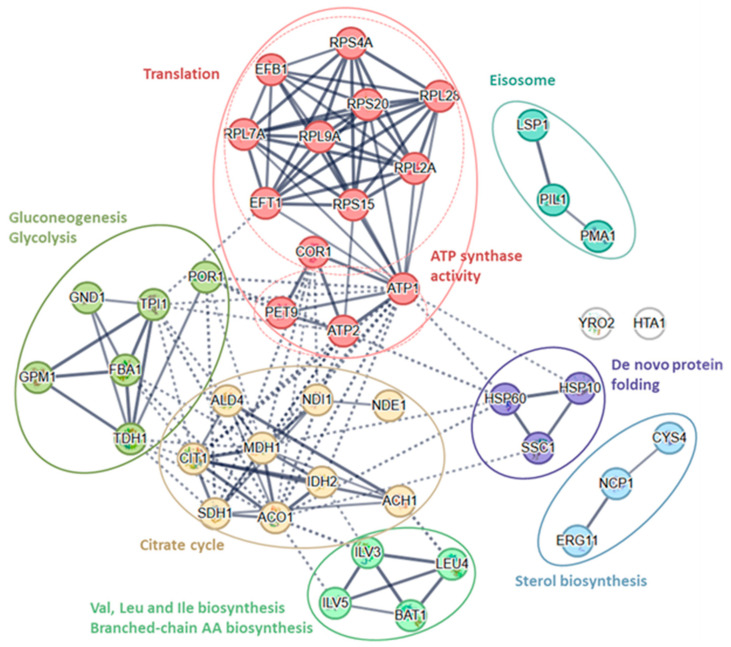
K-means cluster of 43 significantly different proteins from the String database.

All identified proteins (43) were clustered into nine groups by the String database (k-means clustering, Table 3, Figure 3c,d and Figure 4).

The cluster of translation proteins (Figure 4, Table 3) consists of nine proteins (Efb1, Eft1, Rpl2A, Rpl7A, Rpl9A, Rpl28, Rps4A, Rps15, Rps20). There are four proteins of proton-transporting two-sector ATPase complexes (Atp1, Atp2, Cor1, Pet9), six proteins of glycolysis/gluconeogenesis (Fba1, Gnd1, Gpm1, Por1, Tdh1, Tpi1) and nine TCA cycle proteins (Ach1, Aco1, Ald4, Cit1, Idh2, Mdh1, Ndi1, Nde1, Sdh1). Four proteins are involved in the biosynthesis and metabolism of amino acids (Bat1, Ilv3, Ilv5, Leu4), three are involved in de novo protein folding (Hsp10, Hsp60, and Ssc1), are involved in sterol biosynthesis (Cys4, Erg11, Ncp1), and three are involved in the formation of the Eisosome filament (Lsp1, Pil1, and Pma1). There are two non-classified (diverse group of) proteins (Hta1 and Yro2).

The total amount of significantly altered proteins (Table 3) in the control group was 255.5 fmol—it was significantly reduced in sample 3D (129.3 fmol) and 28D (140.9 fmol), while in sample 7D the amount was slightly increased (264.2 fmol) and in 14D significantly increased (315.5 fmol).

Each experimental group has its own profile of grouped proteins by function (Figure 3c,d). In the untreated control group, which can be considered a reference, the metabolic processes for energy supply (glycolysis/gluconeogenesis, TCA cycle, and ATP synthesis) dominated with 50% of the total amount of significantly altered proteins. Although the total amount is almost the same in all samples, there is a significant difference in the distribution of proportions. Interestingly, differences were observed even among the treated samples. In the 14D and 28D samples, the proportion of gluconeogenic/glycolytic proteins increases significantly, primarily due to Glyceraldehyde-3-phosphate dehydrogenase (Tdh), whose levels nearly double compared to the control, while the proportion of TCA cycle enzymes in those samples is reduced. The opposite was with the sample 7D, whose gluconeogenic/glycolytic protein content was the lowest (lowest Tdh), while TCA cycle enzymes were the highest, mainly because of the presence of Idh (subunit of isocitrate dehydrogenase) and two acetate metabolism proteins (aldehyde dehydrogenase, Ald4, and acetyl-CoA hydrolase, Ach1).

The relative amounts of proteins in each experimental group (Figure 3d and Supplementary S2_calculations_2, Table 3) were calculated based on the molar ratio of total proteins within each clustered group. Translation-related proteins accounted for 10.4% of the total protein content in the control sample and showed increased levels in the 14D (23.3%) and 28D (17.2%) samples, while remaining largely unchanged in 3D and 7D. The greatest changes in the amount of proteins in the treated samples were observed in sample 14D, where the amount of translation proteins nearly doubled compared to 3D, 7D, and the control group. Although the fraction of translational proteins (Supplementary S2_calculations_2) primarily represents the cytosolic fraction co-isolated with mitochondria, it is noteworthy because it reflects the transcriptional response of the treated samples to metal ion–induced stress.

It is known that translation begins with the recruitment of the initiation factor of the 40S subunit and the scanning of the start codon. After the release of the initiation factor, the 60S subunit joins the complex to form a functional ribosome. Our results show that the content of both 40S and 60S ribosomal subunits increased, particularly in 3D and 28D samples, whereas in 7D and 14D samples, total subunit content remained similar to the control. While individual levels of large and small subunits appeared unchanged, the 40S:60S ratio shifted significantly: 1:1 in the control, increasing progressively from 2 in 3D, to 2.5 in 7D, 5.3 in 14D, and 5.5 in 28D.

Among all known elongation factors, two showed significant changes. Efb1 (translation elongation factor beta 1), the most abundant in the control sample, was markedly reduced in the treated samples and was undetectable in 3D. The other, Eft1 (elongation factor 2), remained highly abundant in all samples except in 28D, where its levels were notably decreased.

Within the group of branched-chain amino acid (BCAA) and leucine biosynthesis proteins, a slight increase in protein levels was observed only in the 7D sample, while in all other treated samples, levels were significantly lower compared to the control. This reduction is primarily due to the complete silencing of Bat1 (branched-chain-amino-acid aminotransferase) and Leu4 (alpha-isopropylmalate synthase) in the 14D and 28D samples, as well as their downregulation in the 3D sample. The most consistently expressed protein across all samples was Ilv5 (ketol-acid reductoisomerase), which remained the dominant component. Ilv5 is not only involved in BCAA biosynthesis but also functions as a mitochondrial DNA-binding protein, contributing to the maintenance of mitochondrial DNA integrity [36].

## 4. Discussion

Essential metals are necessary for the normal functioning of the body, but at the same time, metal ions can be potentially dangerous for cell survival. It is therefore important that cells initiate appropriate adaptive responses to metal-induced stress. Stress means a major loss of energy resources and forces the cell to adapt at all levels [7,14,30,37,38]. Research has shown that the yeast *S. cerevisiae* is able to compensate for the toxic effect of different metals and survive in various forms of toxic stress [4,11,15,21,26]. In this study, we set the yeast a very challenging task. We exposed them to a mixture of different metal ions in increasing concentrations on complete yeast media under microaerophilic conditions and allowed them to grow to the early stationary phase. The importance of conducting experiments on yeast, specifically in the stationary phase, has been described in detail by Kovač et al. [11]. In this phase, yeast shifts from fermentative to mitochondrial respiration, which closely resembles the normal respiration of multicellular eukaryotes. This transition leads to increased production of ROS and the upregulation of defense and repair mechanisms. The required time to reach that phase was established in previous experiments as approximately 20 h for all media with eluted metal ions used in the study. Exposure to metal ions altered cell growth (Figure 1), and the cell responded differently to increasing amounts and different types of metal ions in the media. Metal ions in growth media inhibit growth, but judging by the number of cells, sample 3D appears to be more toxic than the other samples, even though it contains the lowest total amount of metal ions (approx. 3 mg/L). The total amount of metal ions in the growth medium increased in samples 7D and 14D, as well as the number of cells. The number of cells decreased in sample 28D (compared to 14D), although the total amount of metal ions increased (the values of Al, 1.2 mg/L, Fe, 8.1 mg/L and Ni, 1 mg/L were particularly high). Aulakh et al. explain in their paper that an extensive homeostatic machinery exists in yeast cells, allowing them to buffer cellular metal ion concentrations. Furthermore, they showed that some metals are actually essential and that some metals only in “excess” amounts slow down cell growth. Thus, 0.8 mg/L Cu (CuSO_4_) and 5 mg/L Fe (FeCl_3_) resulted in a slowing of cell growth, indicating toxicity [14]. In our research, sample 28D contained a lower amount of Cu (0.5 mg/L) but a higher amount of Fe. Therefore, the reduction in cell numbers could be due to an excess of Fe or some other metals and their interlinked nature. Hosiner et al. confirmed the toxicity of Al, Mn, and Ni at even lower concentrations than in this research [3].

The ratio of apoptotic and necrotic cells changes in the opposite direction compared to the number of cells. Accordingly, in sample 14D, the number of apoptotic cells is lower than in 3D. Apoptosis in yeast, triggered by ROS as crucial modulators, has been proposed as an altruistic elimination mechanism for old and damaged cells. Those cells release nutrients, increasing the survival chances of younger or healthier clone cells [7]. Fabrizio et al. [39] suggested that apoptosis-associated ROS accumulation increases the probability of clonal mutations in yeast populations, resulting in a greater number of yeast cells that are better adapted to altered growth conditions. It is possible that this phenomenon occurred in the cells in this study (sample 3D–14D).

We can say that the control mechanisms of the stress response are programmed to be activated at a certain stress threshold. They should not be triggered when the stress level is too low, so that they do not often stop the growth and proliferation of cells. On the other hand, they must be activated with sufficient speed and amplitude to prevent cell damage. And finally, there must be a way for the cell to recover from the stress [14,22].

Yeast dynamically changes the composition of its proteome to respond to stress. The reprogramming of gene expression in response to stress usually initially involves a global suppression of protein synthesis. However, against the background of a global down-regulation of protein synthesis, activation of specific stress-responsive mRNAs is also induced by translational and transcriptional responses [23,37,40]. The global suppression of protein synthesis was most noticeable in the 3D sample in this study, where cells respond either with suppression of translation or with silencing of some proteins (Rpl7A, Rps15, Eft1, Efb1). In the 7D and 14D samples, the majority of translational proteins were overexpressed (in 14D samples several times), while in the 28D sample, some proteins were reduced, and some were increased. Initiation factors have been recognized as key sites for the regulation of transcription. Less attention has been paid to other steps in translation, although it is increasingly recognized that cells have evolved regulatory steps to modulate translation under stress that go beyond initiation mechanisms [37,40].

Thus, based on the results of the changes in the group of translation proteins, the initiation factors, the 40S subunits, the 60S subunits, and the multiple elongation factors have changed and fluctuated to support the stress response differently and uniquely in each type of sample. Moreover, for each type of sample, some “peculiarities” were observed in the stress response—either in the amount or in the appearance of a new protein.

Glycolysis and alcoholic fermentation are well-studied metabolic pathways involved in the conversion of sugar. Recent studies on yeast have shown that the most significant glycolytic enzymes are abundant proteins whose expression remains unaffected during the transition from exponential to stationary phase under anaerobic conditions [41]. Based on this, and the fact that in this study the cells grown under microaerophilic conditions were collected in an early stationary phase, all observed changes in the proteins of the Klaster group: glycolysis/gluconeogenesis, ATP synthesis, and TCA cycle were indeed stress response proteins of the yeast.

A common response to OS that occurs in all organisms is the reduction in cellular ATP concentration, which is mainly achieved by the inactivation of key glycolytic enzymes to ensure a “quasi-quiescent” state. This significant decrease in cellular energy content can be attributed to the oxidative inactivation of key glycolytic enzymes responsible for the active detour of glucose from glycolysis into the pentose phosphate pathway, effectively reducing ATP synthesis at the expense of NADPH generation [42]. The majority of antioxidant systems are inevitably based on the ratio of NADPH to NADP^+^. The redirection of glycolytic flux into NADPH-generating processes plays an important role in antioxidant protection at the cellular and organismal levels [43].

The possible reduction in cellular ATP concentration is further reflected in the decreased abundance of Atp1 and Atp2, subunits of the mitochondrial ATP synthase complex essential for oxidative phosphorylation and energy production, as well as Pet9, which functions as the ADP/ATP translocase [18,34]. All these proteins exhibit reduced expression (green in Figure 3a) in 14D and 28D samples. This downregulation may be associated with iron overload observed in these samples, which is known to disrupt mitochondrial function by impairing iron–sulfur cluster biogenesis, mitochondrial protein translation, and by depleting cellular glutathione levels. Such dysregulation compromises the integrity of the respiratory chain and energy metabolism, ultimately leading to reduced ATP production and altered expression of key mitochondrial proteins [44].

One of the regulating glycolytic points is glyceraldehyde-3-phosphate dehydrogenase (Tdh1), which catalyzes the reversible oxidative phosphorylation of G3P to 1,3-bisphosphoglycerate (1,3-BPG) using NAD^+^ and inorganic phosphate. This enzyme is generally present in considerable amounts, with a fairly wide range between groups (the lowest amount in 7D (12.3 fmol) and the highest in 14D (41.3 fmol)) in this work (Table 3). The cysteine in the active site of Tdh1 is very sensitive to inhibitory oxidative modifications by ROS. In addition to direct thiol oxidation by ROS, Tdh1 is rapidly S-thiolated after both endogenous and exogenous OS. S-thiolation is a post-translational modification in which proteins form mixed disulfides with low molecular weight thiols [43]. *S. cerevisiae* knockout strains defective in GSH biosynthesis are unable to restore the enzymatic activity of Tdh1, suggesting that GSH is required for protection against irreversible thiol hyperoxidation. According to Shenton and Grant, several glycolytic and related enzymes are targets of S-thiolation [45]. Among them are fructose 1,6-bisphosphate aldolase (Fba), triose phosphate isomerase (Tpi), aldehyde dehydrogenase (Ald), as well as two transcription factors (Efb and Eft), which were also identified as significantly altered in this study. Protection of Tdh1 and other glycolysis-related proteins from irreversible oxidation by S-thiolation could allow a cell to rapidly resume glycolysis and, thus, ATP production after the stress subsides. Without a sufficiently rapid resumption of ATP synthesis, cell death could be the result [45]. To maintain reduced GSH and prevent intracellular accumulation of ROS, it is essential to generate NADPH. NADPH provides the reducing power required to fuel protein-based antioxidant systems and to recycle oxidized glutathione. The oxidative branch of the pentose phosphate pathway is often a major source of NADPH [43]. In addition, for NAD(P)H recovery, the activity of alcohol dehydrogenase 1 (Adh1) is important. Alcohol dehydrogenases are oxidoreductases that catalyze the reversible oxidation of alcohols to aldehydes or ketones with simultaneous reduction in NAD^+^ or NADP^+^ [46]. In this work, Adh1 was detected in all samples, but the values were not significantly altered. Aldehyde dehydrogenase 4 (Ald4), which was detected in control and 7D samples, and was reduced in sample 28D, is also significant. The aldehyde dehydrogenase superfamily comprises NAD(P)^+^-dependent enzymes that oxidize a broad spectrum of endogenous and exogenous aldehydes to the corresponding carboxylic acids [47].

In the TCA cycle cluster, aconitase (ACO1) was the most prominent protein. The heat map shows a relative decrease in its expression in samples 3D, 14D, and 28D compared to the control, and an increase in sample 7D. However, the absolute quantity (fmol) and molar proportions (Table 3) indicate an increase in all treated samples, with the highest proportion observed in sample 28D. Aconitase is a [4Fe-4S]-containing (de)hydratase that is sensitive to O_2_^−^∙ and readily reacts with ROS in a dose-dependent manner [43].

It is worth mentioning the cystathionine-β-synthase (encoded by *CYS4*) enzyme that catalyzes the first step of the transsulfuration pathway from homocysteine to cystathionine [42]. Cys4 was also detected in this work (samples 3D, 14D, and 28D), indicating an active metabolic pathway. Interestingly, it can be allosterically regulated by effectors such as S-adenosyl-L-methionine [48]. Sam1 (S-adenosyl-L-methionine synthase) was also determined in this work, but not in the same samples as Cys4—in the 3D, in 7D, and 14D (the highest value, 16 fmol, Table 3).

So, yeast cells under oxidative stress redirect glucose metabolism from ATP production toward NADPH generation to support antioxidant defense. Tdh1, a redox-sensitive enzyme, undergoes reversible S-thiolation, which protects it from irreversible oxidation and allows rapid post-stress reactivation. Several other glycolytic enzymes and transcription factors were also identified as S-thiolation targets. Additionally, increased Aco1 levels and the presence of Cys4 and Sam1 suggest broader metabolic adaptations to oxidative conditions. The observed protein changes reflect a coordinated metabolic adaptation to maintain redox balance and ensure survival.

The novelty of this study is the use of solutions with a significantly higher concentration of eluted metal ions, even in yeast complete medium as a protein-sugar medium (whereas in most other studies, it is a kind of artificial saliva), and at a pH of 5.5, which is present in the oral cavity of patients with poor oral hygiene. Using a relatively small volume of eluate allowed us to obtain a concentrated solution of eluted ions, enabling the detection of an intense release of a wide range of ions even in such a small volume. This concentration of eluted ions can be considered cumulative over a period of time in the oral cavity and can probably be eliminated from the body due to the large amount of saliva excreted during the day. On the other hand, for hypersensitive people or those with detoxification problems, it provides valuable information about the total amount of ions.

Much is known about the importance of metal ions, but still not enough. It seems that the commonly established experimental conditions do not adequately reflect the actual state in the cell due to the interlinked nature of metal homeostasis and buffering that occurs in specific organelles [14]. In this study, we employed four distinct solutions characterized by varying types and concentrations of metal ions, which were released through elution rather than dissolution of their respective salts. The eluted metals include both essential elements and those known to exhibit (cyto)toxic effects—namely Al, Cr, Cu, Fe, Mn, and Ni. These ions were present in the eluates at concentrations that increased with prolonged elution time, representing either physiologically relevant or excessive levels. Importantly, the metals were eluted from contemporary orthodontic appliances, which are widely used in clinical practice. The findings of this work provide valuable insights into the potential toxicity associated with these devices and contribute to a deeper understanding of their biocompatibility.

The medium used in this study was also different. It is a yeast complete medium, which is not common in orthodontic studies, but reflects the actual state of the oral cavity better than pure artificial saliva (which would mean nothing is eaten or drunk). In this way, it contains both sugars and proteins, and the effect of such a medium on the metal parts of the orthodontic appliance is different both in terms of the amount and type of metal ions (which is confirmed in [12]).

All experiments were conducted using the same growth medium supplemented with 2% glucose, which is recognized as the primary and preferred carbon source for yeast. Monitoring extracellular and metabolized glucose levels is essential for the regulation and coordination of yeast carbon metabolism [24]. In all samples containing metal ion eluates, glucose consumption occurred within a comparable timeframe, indicating that the isolated proteins likely reflect the cellular response to the presence of metal ions in the substrate. Furthermore, a pronounced reorganization of mitochondrial metabolism was consistently observed across all conditions, underscoring the impact of metal exposure on mitochondrial function and cellular adaptation. Using the simple eukaryote *S. cerevisiae* as a model organism in this study highlights the remarkable adaptability of cells and their ability to deploy diverse strategies to combat environmental stress.

Additionally, a crude mitochondrial fraction was tested intentionally, which can be considered a weakness of the study. However, the intention was to target mitochondria and related proteins metabolically bound to mitochondria and “catch” exactly the part of the proteins that was most active in the stress response. Mitochondria from all yeast samples were isolated on the same day using a standardized Isolation Kit to ensure consistency and comparability across samples. In addition to a wide range of mitochondrial proteins, numerous contaminants—primarily cytosolic and ribosomal proteins—were also detected. Despite being considered contaminants, these proteins significantly contribute to the interpretation of cellular responses to stress, offering valuable insights into the broader proteomic changes.

To further validate the proteomic findings, we plan to perform quantitative PCR (qPCR) analysis of selected genes corresponding to differentially expressed proteins. Although this experiment will be conducted separately, it will serve as an independent cross-validation approach to strengthen the reliability of the observed expression patterns.

## 5. Conclusions

This study aimed to examine the influence of metal ions released from the surface of orthodontic appliances on *S. cerevisiae* W303 cells. The question was how the yeast cells cope with the stress caused by the metal ions. It was found that exposure to metal ions triggers adaptive responses that alter cell growth. The cells reacted differently to increased amounts of metal ions in the medium. The response of the cells can be roughly summarized in the way that they were hierarchically clustered based on the expression patterns. Thus, the control was clustered with the 7D sample, 14D with 28D, and 3D was similar to the 14D and 28D groups.

Exposure to metal ions increases intracellular levels of oxidants, which induce changes in protein structure and function. The stress response triggers a reprogramming of gene expression in the yeast, dynamically altering the composition of the proteome. The study showed that all proteins involved in translation changed in response to the stress and fluctuated differently for each sample type. Since several significantly altered glycolytic and related enzymes are targets of S-thiolation, GSH appears to play an important role in protecting against irreversible thiol hyper-oxidation. Furthermore, the observed changes in glycolytic and TCA cycle enzymes were likely linked to alterations in cellular ATP levels and the cell’s efforts to produce NADPH. Proteins involved in the biosynthesis and metabolism of specific amino acids also changed in ways that help cells recover from oxidative damage. In addition, the downregulation of mitochondrial proteins such as Atp1, Atp2, and Pet9 under conditions of metal (possibly iron) overload suggests a broader impairment of mitochondrial function with disruption of mitochondrial homeostasis. Such dysregulation likely contributes to reduced ATP levels and reflects a systemic metabolic adaptation to sustained oxidative stress.

Although we have attempted to correlate the quality and quantity of the eluted metal ions with the results of the proteome analysis, e.g., in the form of specifically altered proteins or indications of a stronger involvement of a particular metabolic pathway based on the identified proteins or indications of the switching on or off of a metabolic pathway, we were not able to establish this correlation. What we can say with certainty is that there are three levels of response to stress—at relatively low eluted metal ion concentrations in the medium (3D, about 3 mg/L), at medium concentrations (7D, about 5.5 mg/L) and at high, excessive concentrations (>8 mg/L, 14D and 28D), and that the response at each of these levels has its specificity. Although much is known about the molecular targets and responses to OS, many unresolved mechanisms and questions with no reliable answers are still present. We hope that the data provided by this research will contribute to some new answers.

## Figures and Tables

**Table 1:** The most important metal ions in the experimental media used in the study (in μg/L of the medium). A detailed analysis can be found in [12].

μg/L	Al	Cr	Cu	Fe	Mn	Ni
CONTROL	114	9	17	670	24	8
3D	215	55	163	2292	96	303
7D	240	38	294	4482	174	568
14D	585	39	534	5989	153	658
28D	1188	56	531	8083	172	992

**Table 2:** Gradient conditions for the separation of tryptic peptides.

Time (min)	% A (0.1% HCOOH)	% B (0.1% HCOOH + 95% CH_3_CN)
Initial	99.0	1.0
0.10	99.0	1.0
40.0	65.0	35.0
43.0	50.0	50.0
57.0	1.0	99.0
60.0	99.0	1.0
65.0	99.0	1.0

**Table 3:** Absolute quantities (fmol) of 43 significantly altered proteins clustered by STRING-derived K-means values. Those proteins were changed upon treatment of the yeast with orthodontic appliances eluates obtained after 3 days (3D), 7 days (7D), 14 days (14D), and 28 days (28D) of elution and YPD media as control.

Protein (Common Name)	Protein Abbreviation	Quantity (fmols)				
		Control	3D	7D	14D	28D
**Translation proteins**						
Translation elongation factor 1 β	Efb1	3.60	0.00	2.17	0.77	1.74
Elongation factor 2	Eft1	4.74	1.88	5.00	14.12	0.00
Ribosomal 60S subunit protein L28	Rpl28	0.00	3.65	1.37	17.84	5.98
Ribosomal 60S subunit protein L2A	Rpl2A	1.91	0.98	5.81	14.13	5.82
Ribosomal 60S subunit protein L7A	Rpl7A	7.33	2.79	5.53	9.99	4.77
Ribosomal 60S subunit protein L9A	Rpl9A	0.00	0.00	0.71	7.26	2.48
Protein component (PC) of the small (40S) ribosomal subunit	Rps4A	5.80	2.94	4.23	9.27	2.95
PC of the small (40S) ribosomal subunit	Rps15	1.94	<0.01	1.21	0.00	0.52
PC of the small (40S) ribosomal subunit	Rps20	1.18	0.75	0.00	0.00	0.00
	SUM	26.51	12.98	26.04	73.39	24.26
**ATP-synthase activity**						
α-subunit of F1, F1F0 ATP synthase	Atp1	8.00	5.26	8.46	8.89	3.97
β-subunit of F1, F1F0 ATP synthase	Atp2	10.51	7.96	14.60	12.35	6.44
Core subunit of the Cyt-c reductase	Cor1	5.35	3.64	5.70	1.83	2.18
ADP, ATP carrier protein 2	Pet9	4.90	4.02	6.00	4.36	2.47
	SUM	28.76	20.88	34.76	27.43	15.06
**TCA cycle**						
Acetyl-CoA hydrolase	Ach1	3.85	0.00	4.87	0.00	0.00
Aconitate hydratase	Aco1	10.79	5.05	13.01	17.25	8.52
Aldehyde dehydrogenase	Ald4	8.25	0.00	8.14	0.00	0.74
Citrate synthase	Cit1	2.45	1.05	3.69	3.43	2.07
Isocitrate dehydrogenase 2	Idh2	2.59	0.00	2.11	0.00	0.00
Malate dehydrogenase	Mdh1	6.49	2.02	7.18	2.95	1.35
Mitochondrial external NADH dehydrogenase	Nde1	1.69	1.69	2.39	1.18	0.00
NADH/ubiquinone oxidoreductase	Ndi1	2.11	1.72	2.65	2.13	0.69
Flavoprotein subunit of succinate dehydrogenase	Sdh1	3.69	0.64	5.64	2.85	0.78
	SUM	41.89	12.17	49.70	29.79	14.16
**Gluconeogenesis/glycolysis fraction**						
Fructose 1,6-bisphosphate aldolase	Fba1	4.34	4.48	4.60	10.15	4.53
6-phosphogluconate dehydrogenase	Gnd1	2.48	0.59	0.65	3.25	0.85
Phosphoglycerate mutase	Gpm1	7.71	4.02	6.35	13.77	6.63
Mitochondrial porin 1	Por1	18.39	9.95	17.27	14.92	7.40
Glyceraldehyde-3-phosphate dehydrogenase 1	Tdh1	19.58	12.27	15.83	41.32	18.04
Triose phosphate isomerase	Tpi1	7.00	1.47	5.24	16.66	3.95
	SUM	59.51	32.49	49.95	100.06	41.39
**Val, Leu, and Ile biosynthesis and branched-chain amino acid biosynthesis**						
Branched-chain-AA aminotransferase	Bat1	2.82	0.80	4.11	0.00	0.00
Dihydroxyacid dehydratase	Ilv3	3.44	2.27	4.37	1.01	2.11
Acetohydroxyacid reductoisomerase	Ilv5	19.68	9.09	21.75	20.60	8.63
2-isopropylmalate synthase	Leu4	2.45	0.42	2.72	0.00	0.00
	SUM	28.39	12.58	32.94	21.61	10.74
**Eisosome fraction**						
Sphingolipid long-chain base-responsive protein	Lsp1	3.59	1.09	4.04	1.60	1.36
Sphingolipid long-chain base-responsive protein	Pil1	4.91	2.47	5.45	2.15	1.16
P2-type H^+^-ATPase	Pma1	29.05	15.61	25.87	33.12	16.62
	SUM	37.56	19.16	35.35	36.87	22.14
**Sterol biosynthesis**						
Lanosterol 14-alpha-demethylase	Erg11	2.59	0.00	4.79	3.58	1.30
Cystathionine beta-synthase	Cys4	0.00	0.13	0.00	2.39	0.31
NADP-cytochrome P450 reductase	Ncp1	1.19	0.44	1.75	1.95	0.00
	SUM	3.78	0.57	6.54	7.92	1.61
**De novo protein folding**						
Heat shock protein 10	Hsp10	2.12	2.50	1.89	0.00	0.65
Heat shock protein 60	Hsp60	3.07	2.71	4.17	4.07	4.11
Heat shock protein HSP70 family	Ssc1	11.52	6.81	14.06	12.21	6.78
	SUM	16.72	12.03	20.12	16.28	11.53
**Rest**						
Histone H2A	Hta1	8.65	2.07	6.99	2.17	0.00
Protein with a putative role in response to acid stress	Yro2	3.68	4.41	1.80	0.00	0.00
	SUM	12.33	6.48	8.79	2.17	0.00
TOTAL		255.46	129.34	264.20	315.5	140.89

## Data Availability

Raw and processed data are publicly available in Zenodo (https://doi.org/10.5281/zenodo.16998515) and in the DABAR repository (URNs: urn:nbn:hr:184:736108, urn:nbn:hr:184:648022, urn:nbn:hr:184:964136).

## Supplementary Materials

The following supporting information can be downloaded at: https://www.mdpi.com/article/10.3390/microorganisms13092200/s1. Supplementary S1: Volcano plots; Supplementary S2: Hierarchically clustered significant proteins and all related data.
