# Supplementary material for: Proteomic Research of the Stress Response of Saccharomyces cerevisiae W303 Yeast to Metal Ions Eluted from Orthodontic Appliances
Source: Microorganisms. 2025 Sep 19;13(9):2200. doi: 10.3390/microorganisms13092200 (PMC12472195; doi:10.3390/microorganisms13092200)

Figure S1. A representative visualization of the differences in protein expression between two conditions using volcano plots: a) control C, b) sample 3D, c) sample 7D, d) sample 14D and e) sample 28D compared to all other samples. This shows which proteins are significally more or less expressed between two samples. Axis-x represent the difference between two samples (log2 fold change), and axis-y represent the p-value (-log10).

Proteins which are significally downregulated are marked in green (log2 diff < – 1, $p < 0{,}05$), while significally downregulated proteins are shown in red (log2 diff > 1, $p < 0{,}05$). Significant proteins lie above the significance line, while all values below the line are colored gray and are not significant. The closer the value is to the line, the more significant it becomes. Proteins that are further to the right or left side have a greater change in expression, and proteins that are higher in the diagram have a lower p-value ($p < 0.05$) and are statistically more significant.

a)

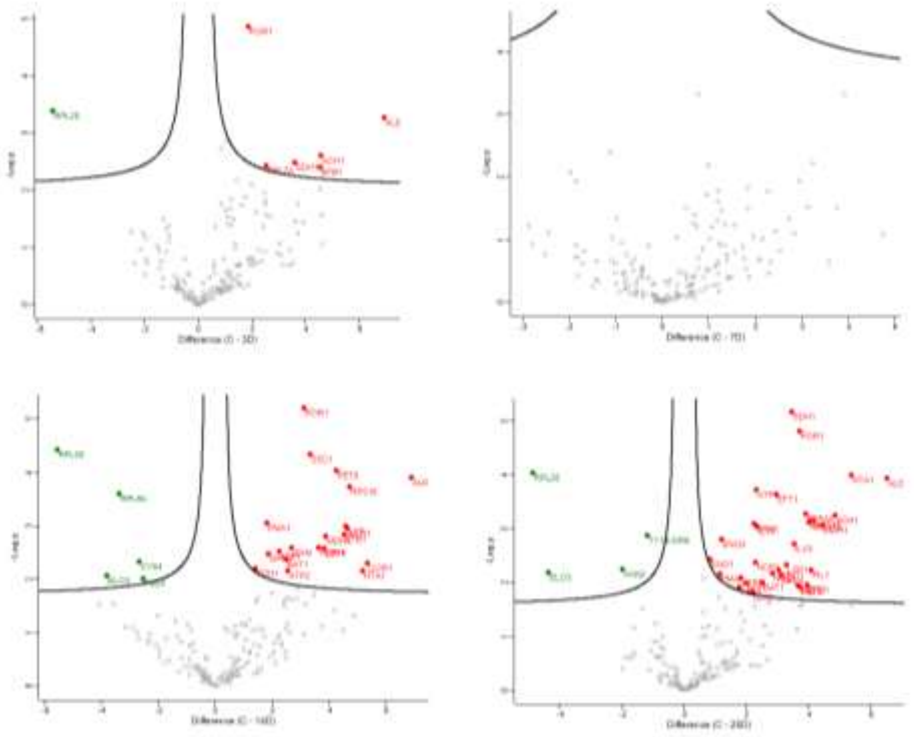

b)

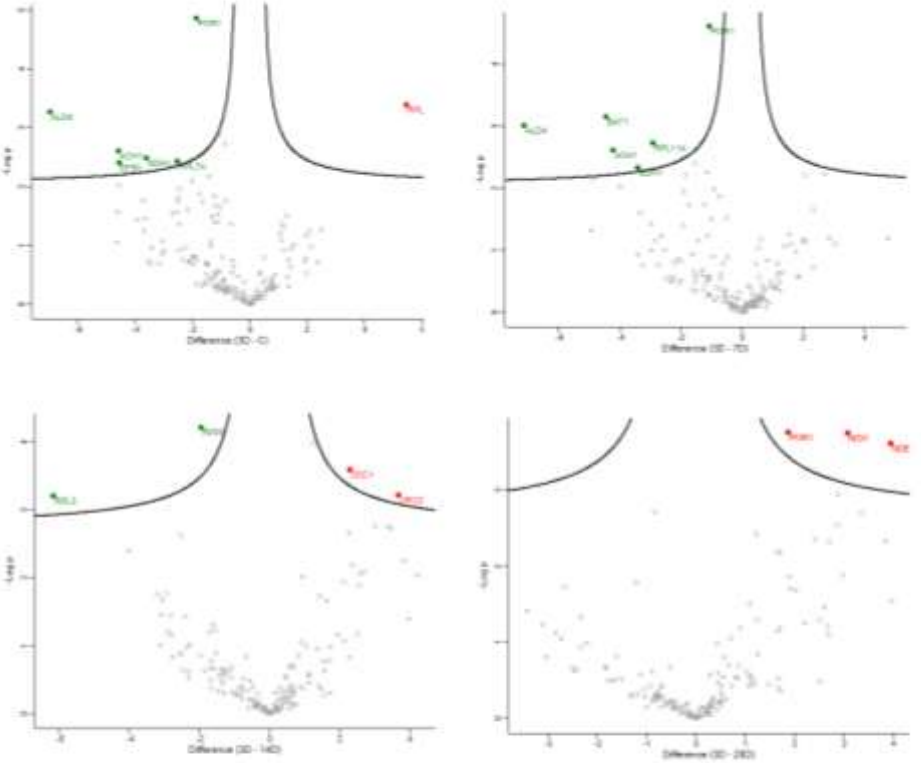

c)

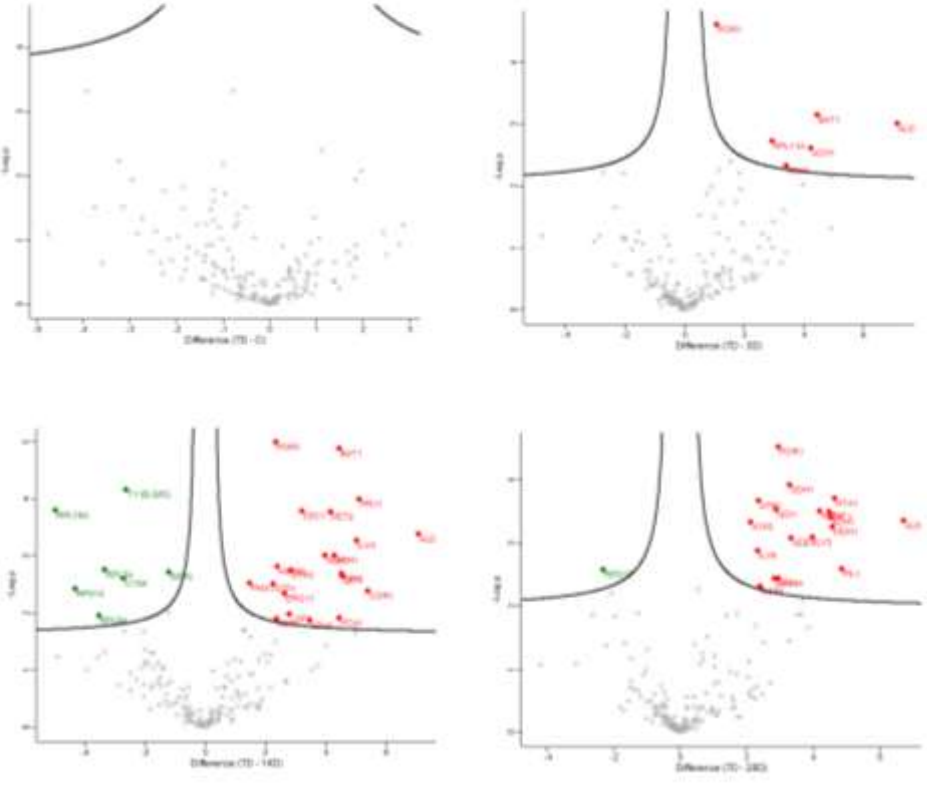

d)

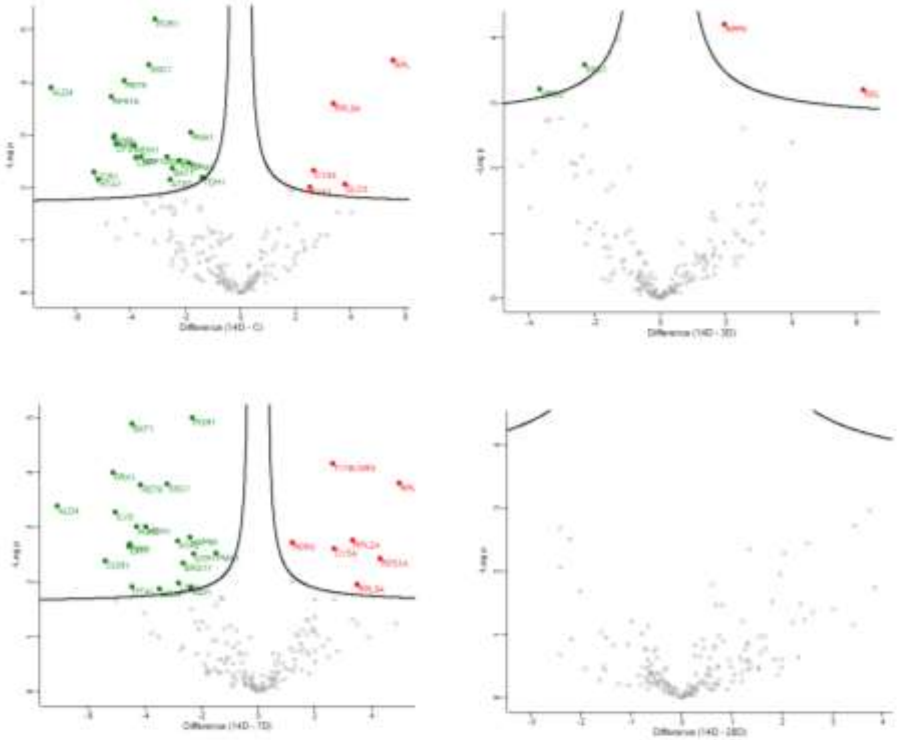

e)

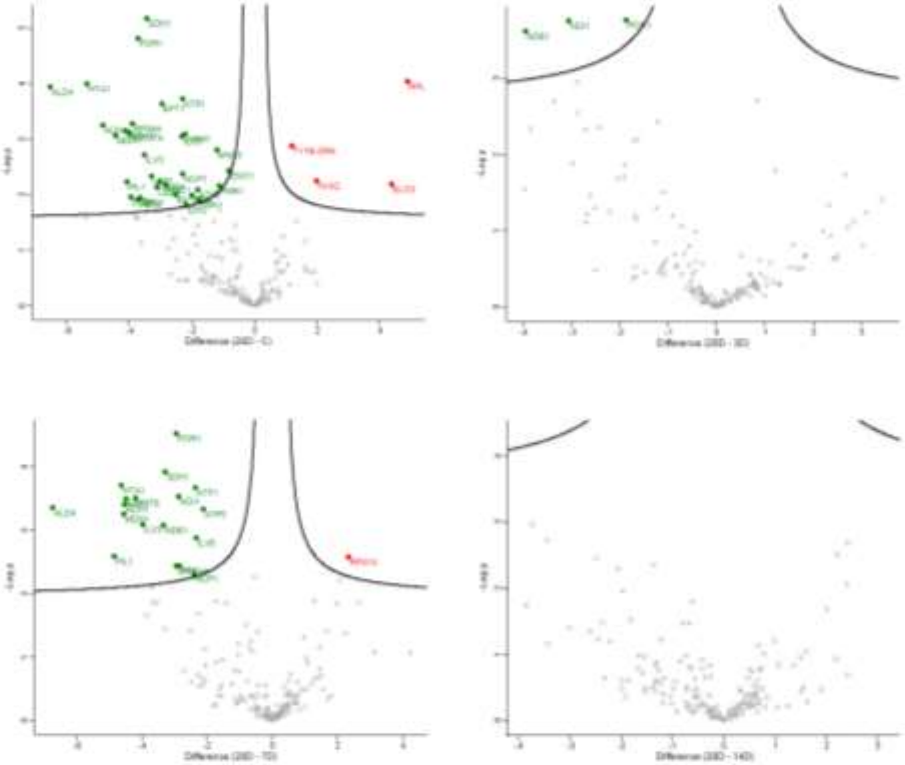

Supplement: Supplementary file 1 [file microorganisms-13-02200-s001.zip › Supplementary S1/Figure - S1 Volcano plots.pdf]
