# Supplementary material for: Proteomic Research of the Stress Response of Saccharomyces cerevisiae W303 Yeast to Metal Ions Eluted from Orthodontic Appliances
Source: Microorganisms. 2025 Sep 19;13(9):2200. doi: 10.3390/microorganisms13092200 (PMC12472195; doi:10.3390/microorganisms13092200)
Supplement: Supplementary file 1 [file microorganisms-13-02200-s001.zip › Supplementary S2/S2_SIGNIFICANT proteins_AMOUNTS AND INTENSITIES.pdf]

SIGNIFICANT PROTEINS

Accession	Entry	gene name	intensity C	intensity 3D	intensity 7D	intensity 14D	intensity 28D	C (fmol)	3D (fmol)	7D (fmol)	14D (fmol)	28D (fmol)	C (ng)2	3D (ng)	7D (ng)	14D (ng)	28D (ng)
P00360	G3P1_YEAST	**TDH1**	43463.12	31225.68	7127.18	16872.04	13963.22	19.5849	12.2708	15.8295	41.3161	18.0378	0.7002	0.4387	0.5659	1.4771	0.6448
P00830	ATPB_YEAST	**ATP2**	58826.62	51079.57	68753.82	10175.26	16130.92	10.5141	7.9576	14.5990	12.3498	6.4442	0.5761	0.4360	0.7999	0.6767	0.3531
P00890	CISY1_YEAST	**CIT1**	14225.07	4284.37	24690.34	1215.97	3379.76	2.4530	1.0543	3.6924	3.4322	2.0744	0.1309	0.0563	0.1970	0.1831	0.1107
P00942	TPIS_YEAST	**TPI1**	13375.69	456.14	5773.66	5105.60	2998.24	6.9990	1.4735	5.2386	16.6555	3.9473	0.1876	0.0395	0.1404	0.4463	0.1058
P00950	PMG1_YEAST	**GPM1**	39498.62	17697.12	18114.86	10886.49	8309.74	7.7107	4.0246	6.3539	13.7668	6.6254	0.2129	0.1111	0.1754	0.3801	0.1829
P02406	RL28_YEAST	**RPL28**	0.00	28861.16	4016.19	28476.04	17967.29	0.0000	3.6496	1.3705	17.8408	5.9840	0.0000	0.0610	0.0229	0.2983	0.1001
P04840	VDAC1_YEAST	**POR1**	237729.64	65778.45	138977.27	27608.90	18122.07	18.3905	9.6532	17.2722	14.9208	7.3980	0.5596	0.2937	0.5256	0.4540	0.2251
P04911	H2A1_YEAST	**HTA1**	35773.61	4680.60	21515.91	1179.81	0.00	8.6505	2.0669	6.9933	2.1667	0.0000	0.1210	0.0289	0.0978	0.0303	0.0000
P05030	PMA1_YEAST	**PMA1**	236875.65	131234.82	188671.17	68402.37	109468.91	29.0526	15.6060	25.8684	33.1161	19.6223	2.8942	1.5547	2.5770	3.2990	1.9548
P05737	RL7A_YEAST	**RPL7A**	59283.34	9924.08	17096.25	32153.76	22941.25	7.3339	2.7864	5.5297	9.9868	4.7742	0.2027	0.0770	0.1528	0.2760	0.1319
P05738	RL9A_YEAST	**RPL9A**	0.00	0.00	358.09	4329.16	824.29	0.0000	0.0000	0.7115	7.2586	2.4781	0.0000	0.0000	0.0153	0.1565	0.0535
P06168	ILV5_YEAST	**ILV5**	311526.67	135415.50	315089.00	117797.62	62594.16	19.6777	9.0942	21.7500	20.5987	8.6333	0.8731	0.4035	0.9650	0.9139	0.3831
P06208	LEU1_YEAST	**LEU4**	4084.39	895.82	6419.09	0.00	0.00	2.4547	0.4235	2.7154	0.0000	0.0000	0.1679	0.0290	0.1857	0.0000	0.0000
P07251	ATPA_YEAST	**ATP1**	90448.66	61509.90	92598.75	19769.14	17991.90	7.9949	5.2611	8.4640	8.8915	3.9690	0.4686	0.3083	0.4960	0.5211	0.2326
P07256	QCR1_YEAST	**COR1**	34027.44	15043.08	34132.22	870.05	3472.21	5.3544	3.6411	5.7015	1.8272	2.1776	0.2689	0.1829	0.2864	0.0918	0.1094
P0CS90	HSP77_YEAST	**SSC1**	67198.92	32662.74	61899.91	6680.22	6911.25	11.5273	6.8186	14.0594	12.2063	6.7750	0.8142	0.4816	0.9930	0.8621	0.4785
P0CX35	RS4A_YEAST	**RPS4A**	14199.16	3642.54	6940.74	2727.80	746.77	5.8007	2.9406	4.2346	9.2721	2.9492	0.1706	0.0865	0.1245	0.2727	0.0867
P0CX45	RL2A_YEAST	**RPL2A**	3255.42	258.29	1480.40	13826.20	5482.48	1.9075	0.9769	5.8143	14.1337	5.8192	0.0523	0.0268	0.1594	0.3874	0.1595
P10614	CP51_YEAST	**ERG11**	4524.52	0.00	12337.15	1725.30	1974.27	2.5874	0.0000	4.7861	3.5785	1.2963	0.1571	0.0000	0.2906	0.2173	0.0787
P14540	ALF_YEAST	**FBA1**	25949.81	56385.75	12813.51	29940.46	7789.96	4.3412	4.4833	4.6043	10.1468	4.5329	0.1720	0.1776	0.1824	0.4020	0.1796
P16603	NCPR_YEAST	**NCP1**	2864.86	479.75	3118.24	1387.19	0.00	1.1922	0.4363	1.7515	1.9499	0.0000	0.0915	0.0335	0.1345	0.1497	0.0000
P17505	MDHM_YEAST	**MDH1**	51071.11	15114.00	53931.31	3599.13	2388.24	6.4918	2.0196	7.1839	2.9451	1.3500	0.2314	0.0720	0.2561	0.1050	0.0481
P18239	ADT2_YEAST	**PET9**	77008.35	34659.80	73022.18	4065.87	7643.83	4.8966	4.0172	5.9987	4.3574	2.4661	0.1686	0.1383	0.2065	0.1500	0.0849
P19414	ACON_YEAST	**ACO1**	51467.30	20941.06	78389.90	31849.66	29086.44	10.7943	5.0520	13.0129	17.2506	8.5242	0.9215	0.4313	1.1109	1.4727	0.7277
P19882	HSP60_YEAST	**HSP60**	10296.78	11515.98	12458.37	2466.60	11136.77	3.0762	2.7063	4.1676	4.0725	4.1079	0.1869	0.1644	0.2532	0.2474	0.2496
P28241	IDH2_YEAST	**IDH2**	3024.40	0.00	936.01	0.00	0.00	2.5925	0.0000	2.1109	0.0000	0.0000	0.1030	0.0000	0.0839	0.0000	0.0000
P32316	ACH1_YEAST	**ACH1**	17240.50	0.00	13309.04	0.00	0.00	3.8473	0.0000	4.8718	0.0000	0.0000	0.2259	0.0000	0.2860	0.0000	0.0000
P32324	EF2_YEAST	**EFT1**	4437.75	2182.00	1929.27	7973.02	0.00	4.7424	1.8753	5.0021	14.1221	0.0000	0.4424	0.1750	0.4667	1.3175	0.0000
P32340	NDI1_YEAST	**NDI1**	3754.14	7428.87	6460.03	1373.16	237.75	2.1138	1.7249	2.6533	2.1301	0.6858	0.1210	0.0987	0.1519	0.1220	0.0393
P32471	EF1B_YEAST	**EFB1**	20438.11	0.00	11177.85	569.39	1609.64	3.6022	0.0000	2.1682	0.7721	1.7366	0.0815	0.0000	0.0491	0.0175	0.0393
P32582	CBS_YEAST	**CYS4**	0.00	188.40	0.00	2751.54	235.03	0.0000	0.1345	0.0000	2.3911	0.3099	0.0000	0.0075	0.0000	0.1339	0.0174
P38079	YRO2_YEAST	**YRO2**	1428.66	7182.55	1340.82	0.00	0.00	3.6795	4.4094	1.7996	0.0000	0.0000	0.1425	0.1707	0.0697	0.0000	0.0000
P38701	RS20_YEAST	**RPS20**	1671.58	587.07	0.00	0.00	0.00	1.1805	0.7461	0.0000	0.0000	0.0000	0.0164	0.0104	0.0000	0.0000	0.0000
P38720	6PGD1_YEAST	**GND1**	6618.98	183.74	213.03	1862.46	213.35	2.4845	0.5874	0.6507	3.2536	0.8461	0.1330	0.0314	0.0348	0.1742	0.0453
P38891	BCA1_YEAST	**BAT1**	4064.90	158.06	14686.39	0.00	0.00	2.8190	0.7956	4.1082	0.0000	0.0000	0.1229	0.0347	0.1791	0.0000	0.0000
P38910	CH10_YEAST	**HSP10**	8679.93	11257.70	3837.48	0.00	876.10	2.1206	2.5027	1.8937	0.0000	0.6503	0.0241	0.0285	0.0215	0.0000	0.0074
P39522	ILV3_YEAST	**ILV3**	16287.48	7311.63	21688.56	389.10	1109.40	3.4429	2.2705	4.3662	1.0133	2.1073	0.2164	0.1427	0.2745	0.0637	0.1325
P40215	NDH1_YEAST	**NDE1**	2825.69	5572.20	3678.94	1959.44	0.00	1.6881	1.6877	2.3936	1.1777	0.0000	0.1060	0.1059	0.1502	0.0739	0.0000
P46367	ALDH4_YEAST	**ALD4**	52396.69	0.00	66509.26	0.00	260.33	8.2457	0.0000	8.1431	0.0000	0.7449	0.4677	0.0000	0.4619	0.0000	0.0423
P53252	PIL1_YEAST	**PIL1**	17685.33	14310.29	29239.74	1720.19	817.72	4.9098	2.4657	5.4490	2.1501	1.1590	0.1883	0.0946	0.2090	0.0825	0.0444
Q00711	SDHA_YEAST	**SDH1**	6861.41	248.72	6169.55	1296.40	218.47	3.6639	0.6364	5.6424	2.8500	0.7810	0.2573	0.0447	0.3963	0.2002	0.0548
Q01855	RS15_YEAST	**RPS15**	10188.21	465.52	3001.76	0.00	278.68	1.9434	0.0014	1.2099	0.0000	0.5234	0.0311	0.0003	0.0194	0.0000	0.0084
Q12230	LSP1_YEAST	**LSP1**	13818.32	2195.39	6951.20	471.43	1269.72	3.5936	1.0923	4.0352	1.6001	1.3554	0.1368	0.0416	0.1536	0.0609	0.0516
