# Supplementary material for: Proteomic Research of the Stress Response of Saccharomyces cerevisiae W303 Yeast to Metal Ions Eluted from Orthodontic Appliances
Source: Microorganisms. 2025 Sep 19;13(9):2200. doi: 10.3390/microorganisms13092200 (PMC12472195; doi:10.3390/microorganisms13092200)

All proteins alphabeticaly			C (fmol)	3D (fmol)	7D (fmol)	14D (fmol)	28D (fmol)
P32316	ACH1_YEAST	**ACH1**	3,8473	0,0000	4,8718	0,0000	0,0000
P19414	ACON_YEAST	**ACO1**	10,7943	5,0520	13,0129	17,2506	8,5242
P46367	ALDH4_YEAS1	**ALD4**	8,2457	0,0000	8,1431	0,0000	0,7449
P07251	ATPA_YEAST	**ATP1**	7,9949	5,2611	8,4640	8,8915	3,9690
P00830	ATPB_YEAST	**ATP2**	10,5141	7,9576	14,5990	12,3498	6,4442
P38891	BCA1_YEAST	**BAT1**	2,8190	0,7956	4,1082	0,0000	0,0000
P00890	CISY1_YEAST	**CIT1**	2,4530	1,0543	3,6924	3,4322	2,0744
P07256	QCR1_YEAST	**COR1**	5,3544	3,6411	5,7015	1,8272	2,1776
P32582	CBS_YEAST	**CYS4**	0,0000	0,1345	0,0000	2,3911	0,3099
P32471	EF1B_YEAST	**EFB1**	3,6022	0,0000	2,1682	0,7721	1,7366
P32324	EF2_YEAST	**EFT1**	4,7424	1,8753	5,0021	14,1221	0,0000
P10614	CP51_YEAST	**ERG11**	2,5874	0,0000	4,7861	3,5785	1,2963
P14540	ALF_YEAST	**FBA1**	4,3412	4,4833	4,6043	10,1468	4,5329
P38720	6PGD1_YEAS1	**GND1**	2,4845	0,5874	0,6507	3,2536	0,8461
P00950	PMG1_YEAST	**GPM1**	7,7107	4,0246	6,3539	13,7668	6,6254
P38910	CH10_YEAST	**HSP10**	2,1206	2,5027	1,8937	0,0000	0,6503
P19882	HSP60_YEAST	**HSP60**	3,0762	2,7063	4,1676	4,0725	4,1079
P04911	H2A1_YEAST	**HTA1**	8,6505	2,0669	6,9933	2,1667	0,0000
P28241	IDH2_YEAST	**IDH2**	2,5925	0,0000	2,1109	0,0000	0,0000
P39522	ILV3_YEAST	**ILV3**	3,4429	2,2705	4,3662	1,0133	2,1073
P06168	ILV5_YEAST	**ILV5**	19,6777	9,0942	21,7500	20,5987	8,6333
P06208	LEU1_YEAST	**LEU4**	2,4547	0,4235	2,7154	0,0000	0,0000
Q12230	LSP1_YEAST	**LSP1**	3,5936	1,0923	4,0352	1,6001	1,3554
P17505	MDHM_YEAS	**MDH1**	6,4918	2,0196	7,1839	2,9451	1,3500
P16603	NCPR_YEAST	**NCP1**	1,1922	0,4363	1,7515	1,9499	0,0000
P40215	NDH1_YEAST	**NDE1**	1,6881	1,6877	2,3936	1,1777	0,0000
P32340	NDI1_YEAST	**NDI1**	2,1138	1,7249	2,6533	2,1301	0,6858
P18239	ADT2_YEAST	**PET9**	4,8966	4,0172	5,9987	4,3574	2,4661
P53252	PIL1_YEAST	**PIL1**	4,9098	2,4657	5,4490	2,1501	1,1590
P05030	PMA1_YEAST	**PMA1**	29,0526	15,6060	25,8684	33,1161	19,6223
P04840	VDAC1_YEAS1	**POR1**	18,3905	9,6532	17,2722	14,9208	7,3980
P02406	RL28_YEAST	**RPL28**	0,0000	3,6496	1,3705	17,8408	5,9840
P0CX45	RL2A_YEAST	**RPL2A**	1,9075	0,9769	5,8143	14,1337	5,8192
P05737	RL7A_YEAST	**RPL7A**	7,3339	2,7864	5,5297	9,9868	4,7742
P05738	RL9A_YEAST	**RPL9A**	0,0000	0,0000	0,7115	7,2586	2,4781
Q01855	RS15_YEAST	**RPS15**	1,9434	0,0014	1,2099	0,0000	0,5234
P38701	RS20_YEAST	**RPS20**	1,1805	0,7461	0,0000	0,0000	0,0000
P0CX35	RS4A_YEAST	**RPS4A**	5,8007	2,9406	4,2346	9,2721	2,9492
Q00711	SDHA_YEAST	**SDH1**	3,6639	0,6364	5,6424	2,8500	0,7810
P0CS90	HSP77_YEAST	**SSC1**	11,5273	6,8186	14,0594	12,2063	6,7750
P00360	G3P1_YEAST	**TDH1**	19,5849	12,2708	15,8295	41,3161	18,0378
P00942	TPIS_YEAST	**TPI1**	6,9990	1,4735	5,2386	16,6555	3,9473
P38079	YRO2_YEAST	**YRO2**	3,6795	4,4094	1,7996	0,0000	0,0000

Atp synthesis + translational

	C (fmol)	3D (fmol)	7D (fmol)	14D (fmol)	28D (fmol)
**ATP1**	7,9949	5,2611	8,4640	8,8915	3,9690
**ATP2**	10,5141	7,9576	14,5990	12,3498	6,4442
**PET9**	4,8966	4,0172	5,9987	4,3574	2,4661
**sum**	28,7601	20,8770	34,7632	27,4259	15,0568
**COR1**	5,3544	3,6411	5,7015	1,8272	2,1776
**EFB1**	3,6022	0,0000	2,1682	0,7721	1,7366
**EFT1**	4,7424	1,8753	5,0021	14,1221	0,0000
**RPL28**	0,0000	3,6496	1,3705	17,8408	5,9840
**RPL2A**	1,9075	0,9769	5,8143	14,1337	5,8192
**RPL7A**	7,3339	2,7864	5,5297	9,9868	4,7742
**RPL9A**	0,0000	0,0000	0,7115	7,2586	2,4781
**RPS15**	1,9434	0,0014	1,2099	0,0000	0,5234
**RPS20**	1,1805	0,7461	0,0000	0,0000	0,0000
**RPS4A**	5,8007	2,9406	4,2346	9,2721	2,9492
**sum**	26,5105	12,9765	26,0407	73,3861	24,2647

gluconeogenesis/glycolisis fraction

	C (fmol)	3D (fmol)	7D (fmol)	14D (fmol)	28D (fmol)
**FBA1**	4,3412	4,4833	4,6043	10,1468	4,5329
**GND1**	2,4845	0,5874	0,6507	3,2536	0,8461
**GPM1**	7,7107	4,0246	6,3539	13,7668	6,6254
**POR1**	18,3905	9,6532	17,2722	14,9208	7,3980
**TDH1**	19,5849	12,2708	15,8295	41,3161	18,0378
**TPI1**	6,9990	1,4735	5,2386	16,6555	3,9473
	59,5109	32,4928	49,9492	100,0595	41,3874

	Control	3D	7D	14D	28D
sum all	255,4560	129,3439	264,2010	315,5003	140,8862
	Control	3D	7D	14D	28D
**translation**	10,37771	10,03253	9,856385	23,26024	17,22293
ATP syntha	11,25834	16,14072	13,15785	8,692819	10,68723
**citrate cycle**	16,39829	9,412764	18,81309	9,440753	10,05092
**gluconeogen/glycolisis**	23,29597	25,12128	18,90576	31,71454	29,37649
**AA synthesis**	11,11516	9,728976	12,4677	6,850063	7,623598
**eisosome**	14,70157	14,8164	13,38094	11,68501	15,71246
**sterol biosynthesis**	1,479537	0,441278	2,474505	2,510141	1,140116
**de novo biosyntesis**	6,546777	9,298959	7,615692	5,159679	8,186251
**others**	4,826664	5,007092	3,328085	0,686751	0
	100	100	100	100	100
	50,95259	50,67476	50,87669	49,84811	50,11464

citrate cycle

	C (fmol)	3D (fmol)	7D (fmol)	14D (fmol)	28D (fmol)
**ACH1**	3,8473	0,0000	4,8718	0,0000	0,0000
**ACO1**	10,7943	5,0520	13,0129	17,2506	8,5242
**ALD4**	8,2457	0,0000	8,1431	0,0000	0,7449
**CIT1**	2,4530	1,0543	3,6924	3,4322	2,0744
**IDH2**	2,5925	0,0000	2,1109	0,0000	0,0000
**MDH1**	6,4918	2,0196	7,1839	2,9451	1,3500
**NDE1**	1,6881	1,6877	2,3936	1,1777	0,0000
**NDI1**	2,1138	1,7249	2,6533	2,1301	0,6858
**SDH1**	3,6639	0,6364	5,6424	2,8500	0,7810
sum	41,8904	12,1748	49,7044	29,7856	14,1604

AA synthesis

	C (fmol)	3D (fmol)	7D (fmol)	14D (fmol)	28D (fmol)
**BAT1**	2,8190	0,7956	4,1082	0,0000	0,0000
**ILV3**	3,4429	2,2705	4,3662	1,0133	2,1073
**ILV5**	19,6777	9,0942	21,7500	20,5987	8,6333
**LEU4**	2,4547	0,4235	2,7154	0,0000	0,0000
	28,3943	12,5838	32,9398	21,6120	10,7406

**translation**	9
ATP syntha	4
**citrate cycle**	9
**gluconeogenesis/glycolisis**	6
**AA synthesis**	4
**eisosome**	3
**sterol biosynthesis**	3
**de novo**	3
**others**	2
	43

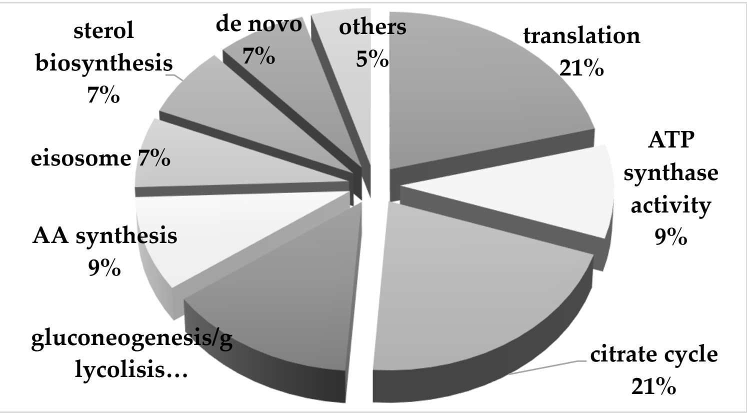

eisosome fraction

	C (fmol)	3D (fmol)	7D (fmol)	14D (fmol)	28D (fmol)
**LSP1**	3,5936	1,0923	4,0352	1,6001	1,3554
**PIL1**	4,9098	2,4657	5,4490	2,1501	1,1590
**PMA1**	29,0526	15,6060	25,8684	33,1161	19,6223
	37,5560	19,1641	35,3526	36,8662	22,1367

sterol biosynthesis

	C (fmol)	3D (fmol)	7D (fmol)	14D (fmol)	28D (fmol)
**ERG11**	2,5874	0,0000	4,7861	3,5785	1,2963
**CYS4**	0,0000	0,1345	0,0000	2,3911	0,3099
**NCP1**	1,1922	0,4363	1,7515	1,9499	0,0000
	3,7796	0,5708	6,5377	7,9195	1,6063

rest	C (fmol)	3D (fmol)	7D (fmol)	14D (fmol)	28D (fmol)
**HTA1**	8,6505	2,0669	6,9933	2,1667	0,0000
**YRO2**	3,6795	4,4094	1,7996	0,0000	0,0000
	12,3300	6,4764	8,7928	2,1667	0,0000

de novo proteins

	C (fmol)	3D (fmol)	7D (fmol)	14D (fmol)	28D (fmol)
**HSP10**	2,1206	2,5027	1,8937	0,0000	0,6503
**HSP60**	3,0762	2,7063	4,1676	4,0725	4,1079
**SSC1**	11,5273	6,8186	14,0594	12,2063	6,7750
	16,7241	12,0276	20,1207	16,2788	11,5333

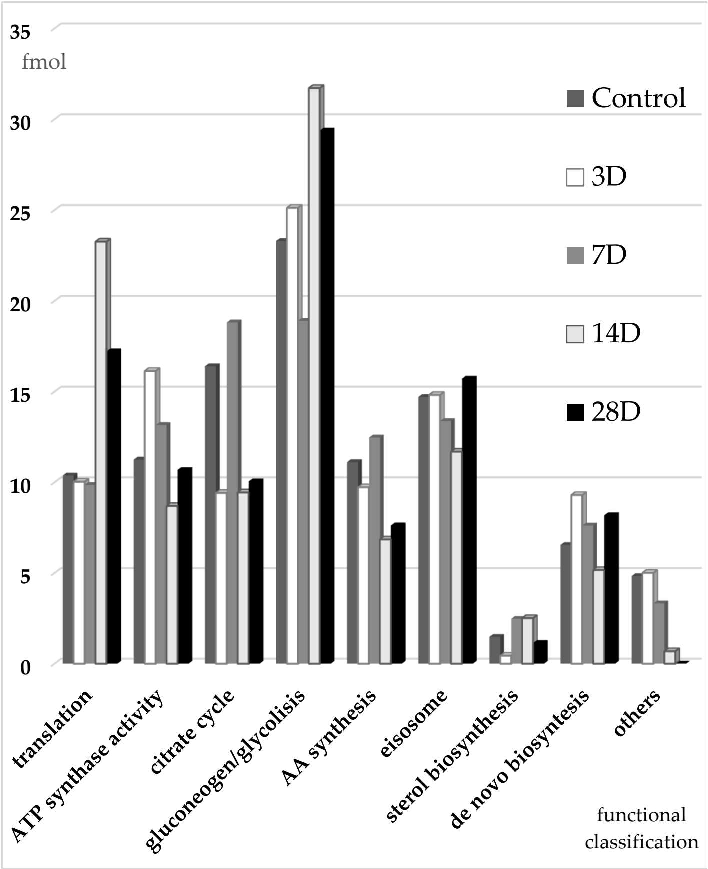

Supplement: Supplementary file 1 [file microorganisms-13-02200-s001.zip › Supplementary S2/S2_SIGNIFICANT proteins_calculations2.pdf]
