# Supplementary material for: Proteomic Research of the Stress Response of Saccharomyces cerevisiae W303 Yeast to Metal Ions Eluted from Orthodontic Appliances
Source: Microorganisms. 2025 Sep 19;13(9):2200. doi: 10.3390/microorganisms13092200 (PMC12472195; doi:10.3390/microorganisms13092200)
Supplement: Supplementary file 1 [file microorganisms-13-02200-s001.zip › Supplementary S2/S2_SIGNIFICANT proteins_HEAT MAP (2).pdf]

	28D	14D	3D	7D	C
**RPL2A**	0,6156232	1,4365143	-0,9983464	-0,5793421	-0,474449
**CYS4**	-0,3597445	1,7841394	-0,3910979	-0,5342753	-0,4990217
**RPL9A**	0,2082859	1,6388345	-0,3217557	-0,8070831	-0,7182816
**RPL28**	0,5728499	0,819802	0,7803339	-0,9641964	-1,2087893
**RPL7A**	0,0180511	0,3705564	-1,2639444	-0,5323056	1,4076425
**TPI1**	-0,4462111	0,3185114	-1,4744661	0,4532841	1,1488818
**HSP60**	0,4379669	-1,7699717	0,496958	0,6172307	0,2178161
**NDI1**	-1,213627	-0,8642111	1,0015849	0,8527024	0,2235509
**NDE1**	-1,624181	-0,1644819	0,9760427	0,5677823	0,2448379
**YRO2**	-0,6030004	-0,8272701	1,6995152	-0,2517042	-0,0175406
**HSP10**	-0,9948329	-0,9301321	1,0873234	-0,1343825	0,9720241
**ACO1**	-0,4819296	-0,3086499	-1,1842347	1,3874831	0,5873311
**ERG11**	-0,4079485	-0,0552317	-1,3138216	1,4011084	0,3758934
**EFB1**	-0,4898294	-0,7777932	-0,7916257	0,5778598	1,4813884
**RPS15**	-0,4779708	-0,7587676	-0,7041534	0,3315198	1,609372
**IDH2**	-0,3280927	-0,7558723	-0,7798432	0,2333462	1,6304621
**GPM1**	-1,0608636	-0,6252161	-0,0361851	0,1542851	1,5679796
**ALD4**	-0,6597368	-0,7572148	-0,7715677	1,1192403	1,0692791
**ACH1**	-0,7996563	-0,7061157	-0,6805358	1,0266308	1,159677
**SDH1**	-0,858791	-0,325702	-0,9427357	1,0131419	1,1140867
**NCP1**	-1,2703097	-0,097903	-0,6235219	1,043013	0,9487216
**LEU4**	-0,9709898	-0,8488824	-0,2783172	1,2192676	0,8789219
**LSP1**	-0,7343971	-1,0743359	-0,2169506	0,6905852	1,3350984
**HTA1**	-0,94892	-0,8739945	-0,2810536	0,9047557	1,1992124
**ILV5**	-1,3074858	-0,4765836	-0,2227703	1,0065343	1,0003054
**RPS4A**	-1,329348	-0,4829283	-0,052643	0,5733768	1,2915425
**POR1**	-1,1788235	-0,7824822	0,0253528	0,7194838	1,216469
**BAT1**	-0,6974724	-0,6802019	-0,6809653	1,5131304	0,5455092
**CIT1**	-0,3537298	-1,2566538	-0,3744343	1,2971585	0,6876594
**PMA1**	-0,4414573	-1,3936944	-0,0350139	0,6976529	1,1725127
**PIL1**	-1,0544314	-1,1101211	0,5533351	0,9662555	0,644962
**MDH1**	-1,1599144	-0,8843936	0,1169129	0,9896982	0,9376968
**PET9**	-0,9304887	-1,188226	0,3189104	0,8780155	0,9217888
**SSC1**	-1,1526942	-0,9754048	0,3375507	0,8571326	0,9334157
**ATP1**	-1,0997747	-1,0380844	0,3576382	0,9038639	0,8763569
**ILV3**	-0,7998478	-1,2840519	0,2914799	0,9964831	0,7959366
**COR1**	-0,724575	-1,3671539	0,3840613	0,8663644	0,8413032
**ATP2**	-0,7724848	-1,3518283	0,5360632	0,9107329	0,6775171
**EFT1**	-1,477798	1,1619576	-0,03973	-0,2700842	0,6256546
**FBA1**	-1,2418741	0,4592321	1,2528683	-0,7673814	0,2971552
**GND1**	-0,5763033	0,3300489	-0,2231055	-1,068972	1,5383319
**RPS20**	-0,268069	-0,726344	0,5647309	-1,0077237	1,4374058
**TDH1**	-0,5315828	-0,1184266	0,6969926	-1,291658	1,2446748

Legend:	28D	14D	3D	7D	C
from -2 to -1.5	***1***	***1***	***0***	***0***	***0***
from -1.5 to -1	***12***	***9***	***4***	***3***	***1***
from -1 to -0.5	***15***	***16***	***9***	***6***	***1***
from -0.5 to 0	***10***	***8***	***12***	***3***	***3***
from 0 to 0.5	***3***	***4***	***8***	***4***	***5***
from 0.5 to 1	***2***	***1***	***6***	***17***	***15***
from 1 to 1.5	***0***	***2***	***3***	***9***	***14***
from 1.5 to 2	***0***	***2***	***1***	***1***	***4***
sum	*43*	*43*	*43*	*43*	*43*
