# Supplementary material for: Proteomic Research of the Stress Response of Saccharomyces cerevisiae W303 Yeast to Metal Ions Eluted from Orthodontic Appliances
Source: Microorganisms. 2025 Sep 19;13(9):2200. doi: 10.3390/microorganisms13092200 (PMC12472195; doi:10.3390/microorganisms13092200)
Supplement: Supplementary file 1 [file microorganisms-13-02200-s001.zip › Supplementary S2/S2_SIGNIFICANT proteins_HYEARCHICAL CLUSTERING.pdf]

SIGNIFICANT PROTEINS hierarhical clustering

Accession	Entry	gene name	C	3D	7D	14D	28D	(-Log) ANOVA p-value	ANOVA q-value	Std. dev.
P00360	G3P1_YEAST	**TDH1**	1,244674802	0,696992636	-1,291658044	-0,118426636	-0,531582773	2,718572053	0,007703704	1,027358422
P00830	ATPB_YEAST	**ATP2**	0,677517056	0,536063194	0,910732865	-1,351828337	-0,772484839	4,276372777	0,002666667	1,258224995
P00890	CISY1_YEAST	**CIT1**	0,687659383	-0,374434263	1,297158480	-1,256653786	-0,353729784	2,464416635	0,012827586	1,783202127
P00942	TPIS_YEAST	**TPI1**	1,148881793	-1,474466085	0,453284144	0,318511367	-0,446211129	1,953809776	0,0344	1,730091663
P00950	PMG1_YEAST	**GPM1**	1,567979574	-0,036185063	0,154285118	-0,625216067	-1,060863614	2,100017431	0,026625	0,849048205
P02406	RL28_YEAST	**RPL28**	-1,208789349	0,780333936	-0,964196444	0,819801986	0,572849929	2,836541322	0,006666667	2,73805393
P04840	VDAC1_YEAST	**POR1**	1,216469049	0,025352824	0,719483793	-0,782482207	-1,178823471	1,025310007	0	1,554382717
P04911	H2A1_YEAST	**HTA1**	1,199212432	-0,281053603	0,904755712	-0,873994529	-0,948920012	2,833239785	0,0064	2,500256621
P05030	PMA1_YEAST	**PMA1**	1,172512650	-0,035013903	0,697652876	-1,393694401	-0,441457272	4,296709269	0,0025	0,706264721
P05737	RL7A_YEAST	**RPL7A**	1,407642484	-1,263944387	-0,532305598	0,370556444	0,018051147	2,064383934	0,027515152	0,948686514
P05738	RL9A_YEAST	**RPL9A**	-0,718281627	-0,321755737	-0,807083070	1,638834476	0,208285928	2,116116663	0,026322581	1,43603066
P06168	ILV5_YEAST	**ILV5**	1,000305414	-0,222770318	1,006534338	-0,476583600	-1,307485819	3,595733195	0,002133333	1,00988505
P06208	LEU1_YEAST	**LEU4**	0,878921866	-0,278317183	1,219267607	-0,848882437	-0,970989823	2,256238062	0,018133333	1,683980774
P07251	ATPA_YEAST	**ATP1**	0,876356900	0,357638240	0,903863907	-1,038084388	-1,099774718	4,530577467	0,001714286	1,169081342
P07256	QCR1_YEAST	**COR1**	0,841303170	0,384061337	0,866364360	-1,367153883	-0,724574983	3,430198878	0,003058824	2,418630702
P0CS90	HSP77_YEAST	**SSC1**	0,933415711	0,337550700	0,857132554	-0,975404799	-1,152694225	4,087103279	0,002909091	1,751455719
P0CX35	RS4A_YEAST	**RPS4A**	1,291542530	-0,052642964	0,573376775	-0,482928336	-1,329347968	3,200582938	0,004	1,491974434
P0CX45	RL2A_YEAST	**RPL2A**	-0,474449009	-0,998346388	-0,579342127	1,436514258	0,615623236	1,891980589	0,039666667	1,652671287
P10614	CP51_YEAST	**ERG11**	0,375893444	-1,313821554	1,401108384	-0,055231743	-0,407948524	1,833697028	0,044864865	1,808478336
P14540	ALF_YEAST	**FBA1**	0,297155172	1,252868295	-0,767381430	0,459232062	-1,241874099	2,808783088	0,006615385	1,152192423
P16603	NCPR_YEAST	**NCP1**	0,948721647	-0,623521924	1,043012977	-0,097902969	-1,270309687	2,949642621	0,00573913	1,037820682
P17505	MDHM_YEAST	**MDH1**	0,937696815	0,116912931	0,989698231	-0,884393573	-1,159914374	5,519348725	0	2,122034298
P18239	ADT2_YEAST	**PET9**	0,921788812	0,318910420	0,878015518	-1,188225985	-0,930488706	4,159191102	0,0032	2,011840975
P19414	ACON_YEAST	**ACO1**	0,587331116	-1,184234738	1,387483120	-0,308649898	-0,481929570	2,60856226	0,009285714	0,743553156
P19882	HSP60_YEAST	**HSP60**	0,217816144	0,496958047	0,617230713	-1,769971728	0,437966853	3,573415179	0,0025	1,003647049
P28241	IDH2_YEAST	**IDH2**	1,630462050	-0,779843152	0,233346194	-0,755872309	-0,328092724	1,827215386	0,044631579	1,117881644
P32316	ACH1_YEAST	**ACH1**	1,159677029	-0,680535793	1,026630759	-0,706115663	-0,799656332	5,335403726	0	2,481084675
P32324	EF2_YEAST	**EFT1**	0,625654638	-0,039729986	-0,270084202	1,161957622	-1,477797985	3,624236319	0,002285714	1,414936785
P32340	NDI1_YEAST	**NDI1**	0,223550856	1,001584888	0,852702439	-0,864211082	-1,213626981	3,925249632	0,002666667	1,386097236
P32471	EF1B_YEAST	**EFB1**	1,481388450	-0,791625738	0,577859819	-0,777793169	-0,489829361	1,800921614	0,045846154	2,004580197
P32582	CBS_YEAST	**CYS4**	-0,499021739	-0,391097873	-0,534275293	1,784139395	-0,359744549	2,992586953	0,005333333	1,165417971
P38079	YRO2_YEAST	**YRO2**	-0,017540559	1,699515224	-0,251704216	-0,827270091	-0,603000402	1,795476995	0,045	1,457704222
P38701	RS20_YEAST	**RPS20**	1,437405825	0,564730883	-1,007723689	-0,726344049	-0,268068999	1,98855814	0,032588235	1,323404472
P38720	6PGD1_YEAST	**GND1**	1,538331866	-0,223105475	-1,068971992	0,330048859	-0,576303303	1,762160551	0,046571429	1,44035391
P38891	BCA1_YEAST	**BAT1**	0,545509219	-0,680965304	1,513130426	-0,680201888	-0,697472394	5,283066903	0	2,028282316
P38910	CH10_YEAST	**HSP10**	0,972024143	1,087323427	-0,134382486	-0,930132091	-0,994832933	1,72367587	0,04855814	1,904353142
P39522	ILV3_YEAST	**ILV3**	0,795936584	0,291479886	0,996483147	-1,284051895	-0,799847782	5,786626916	0	2,2095949
P40215	NDH1_YEAST	**NDE1**	0,244837865	0,976042688	0,567782342	-0,164481878	-1,624181032	2,985370362	0,005454545	1,520703614
P46367	ALDH4_YEAST	**ALD4**	1,069279075	-0,771567702	1,119240284	-0,757214844	-0,659736753	6,49822608	0	3,775150552
P53252	PIL1_YEAST	**PIL1**	0,644961953	0,553335071	0,966255486	-1,110121131	-1,054431438	3,076111002	0,0054	2,399466567
Q00711	SDHA_YEAST	**SDH1**	1,114086747	-0,942735672	1,013141870	-0,325701952	-0,858790994	3,829936794	0,002461538	1,752603018
Q01855	RS15_YEAST	**RPS15**	1,609372020	-0,704153359	0,331519753	-0,758767605	-0,477970809	1,775626731	0,046341463	1,98831262
Q12230	LSP1_YEAST	**LSP1**	1,335098386	-0,216950595	0,690585196	-1,074335933	-0,734397054	3,345711753	0,003777778	1,589236596
